# *Lactobacillus johnsonii* N6.2 Modulates the Host Immune Responses: A Double-Blind, Randomized Trial in Healthy Adults

**DOI:** 10.3389/fimmu.2017.00655

**Published:** 2017-06-12

**Authors:** Guillermo E. Marcial, Amanda L. Ford, Michael J. Haller, Salvador A. Gezan, Natalie A. Harrison, Dan Cai, Julie L. Meyer, Daniel J. Perry, Mark A. Atkinson, Clive H. Wasserfall, Timothy Garrett, Claudio F. Gonzalez, Todd M. Brusko, Wendy J. Dahl, Graciela L. Lorca

**Affiliations:** ^1^Department of Microbiology and Cell Science, Genetics Institute, Institute of Food and Agricultural Sciences, University of Florida, Gainesville, FL, United States; ^2^Food Science and Human Nutrition Department, Institute of Food and Agricultural Sciences, University of Florida, Gainesville, FL, United States; ^3^Department of Pediatrics, College of Medicine, University of Florida, Gainesville, FL, United States; ^4^School of Forest Resources and Conservation, Institute of Food and Agricultural Sciences, University of Florida, Gainesville, FL, United States; ^5^Department of Soil and Water Science, Institute of Food and Agricultural Sciences, University of Florida, Gainesville, FL, United States; ^6^Department of Pathology, Immunology, and Laboratory Medicine, College of Medicine, University of Florida, Gainesville, FL, United States

**Keywords:** *Lactobacillus johnsonii*, diabetes type I, probiotic, indoleamine-2,3-dioxygenase, microbiome, gastrointestinal symptom, T cell, immunological response

## Abstract

*Lactobacillus johnsonii* N6.2 mitigates the onset of type 1 diabetes (T1D) in biobreeding diabetes-prone rats, in part, through changes in kynurenine:tryptophan (K:T) ratios. The goal of this pilot study was to determine the safety, tolerance, and general immunological response of *L. johnsonii* N6.2 in healthy subjects. A double-blind, randomized clinical trial in 42 healthy individuals with no known risk factors for T1D was undertaken to evaluate subject responses to the consumption of *L. johnsonii* N6.2. Participants received 1 capsule/day containing 10^8^ colony-forming units of *L. johnsonii* N6.2 or placebo for 8 weeks. Comprehensive metabolic panel (CMP), leukocyte subpopulations by complete blood count (CBC) and flow cytometry, serum cytokines, and relevant metabolites in the indoleamine-2,3-dioxygenase pathway were assessed. *L. johnsonii* N6.2 survival and intestinal microbiota was analyzed. Daily and weekly questionnaires were assessed for potential effects of probiotic treatment on general wellness. The administration of *L. johnsonii* N6.2 did not modify the CMP or CBC of participants suggesting general safety. In fact, *L. johnsonii* N6.2 administration significantly decreased the occurrence of abdominal pain, indigestion, and cephalic syndromes. As predicted, increased serum tryptophan levels increased resulting in a decreased K:T ratio was observed in the *L. johnsonii* N6.2 group. Interestingly, immunophenotyping assays revealed that monocytes and natural killer cell numbers were increased significantly after washout (12 weeks). Moreover, an increase of circulating effector Th1 cells (CD45RO^+^CD183^+^CD196^−^) and cytotoxic CD8^+^ T cells subset was observed in the *L. johnsonii* N6.2 group. Consumption of *L. johnsonii* N6.2 is well tolerated in adult control subjects, demonstrates systemic impacts on innate and adaptive immune populations, and results in a decreased K:T ratio. These data provide support for the safety and feasibility of using *L. johnsonii* N6.2 in prevention trials in subjects at risk for T1D.

Trial registration: This trial was registered at http://clinicaltrials.gov as NCT02349360.

## Introduction

Commensal bacteria regulate a myriad of host processes and provide several nutrients to their host as well as their symbionts within the microbial community (1, 2). In healthy individuals, these relationships are thought to occur in equilibrium (3). However, disruption of this equilibrium may contribute to various conditions including inflammatory bowel disease and atopy [for a review, see Ref. (4)]. As a result of the multiple microbiome studies being performed, this connection has gained credibility as associations between gut microbiota and either the risk for or presence of a variety of specific human diseases have been shown [for a review, see Ref. (5–7)]. While genetics has been demonstrated to represent a major risk factor for the development of type 1 diabetes (T1D), numerous environmental factors have been suggested that could further elicit a break in immunological tolerance and initiate or perpetuate β-cell autoimmunity (8, 9). Recent studies have looked at the fluctuations in the microbiota and the rate of diabetes development in infant cohorts. These studies have shown a low abundance of lactate-producing and butyrate-producing species and an increase of the *Bacteroides* genus in children with autoimmunity when compared to controls (10, 11). Kostic et al. (12) further showed that the fluctuations in the microbiota composition occur prior to the onset of disease but after seroconversion.

Interactions between the intestinal environment, epithelial barrier function, and the immune system have all been shown to have a major impact on the rate of T1D development in rodent models (13–15). In order to understand the role of the resident microbiota in T1D, we performed a culture-independent analysis of the bacteria in fecal samples collected from biobreeding diabetes-resistant (BB-DR) and diabetes-prone (BB-DP) rats. These experiments demonstrated a significant difference in *Lactobacillus* and *Bifidobacterium* species in the intestinal microbiota of DR and the DP rats, which were correlated with health status (16). Members of these bacterial genera are widely used in dietary supplements as probiotics worldwide. However, the mechanisms by which these individual probiotics modulate host responses and immunity are diverse and are often strain specific, rather than shared among genera (17, 18).

Given the observation of *L. johnsonii* N6.2 in protected DR rats, we performed an intervention study using *L. johnsonii* N6.2 in BB-DP animals. It was found that the administration of *L. johnsonii* N6.2 to BB-DP rats reduced the incidence of T1D (14). The feeding of this microorganism postweaning was followed by changes in the native microbiota, host mucosal proteins, and oxidative stress response. In the ileum, lower levels of the pro-inflammatory cytokines IFN-γ and TNF-α were also observed in the *L. johnsonii* fed group. *L. johnsonii*-mediated diabetes prevention correlated with a Th17 cell bias and elevated IL-23 levels within the mesenteric lymph nodes. Further *in vitro* studies indicate that the modification of dendritic cells (DCs) by oral feeding of *L. johnsonii* N6.2 contributed to the Th17 bias (15).

One potential mechanism by which the host microbial composition may alter immune responses is through the metabolism of tryptophan. This essential amino acid acts as a substrate for the enzyme indoleamine-2,3-dioxygenase-1 (IDO), which converts tryptophan to kynurenine (19–21). T helper subset activation and differentiation has been demonstrated to depend on the bioavailability of local tryptophan (22), and seminal studies showed that murine IDO expression was necessary for T cell tolerance during pregnancy to the semi-allogeneic fetus (23, 24). In an *in vivo* feeding assay performed in BB-DP rats, *L. johnsonii* N6.2 lowered intestinal IDO gene transcription, which in turn correlated with decreased blood plasma kynurenine levels (25). During *in vitro* studies, *L. johnsonii* N6.2 produced H_2_O_2_ that strongly inhibited IDO activity. Mass spectrometry analysis of the IDO catalytic heme-center supported the presence of a molecule in the *L. johnsonii* culture cell-free supernatant that modifies this immunoregulatory enzyme’s prosthetic group, and as a consequence, its activity. These data suggest that this bacterium alters host IDO activity, with the potential for downstream effects on T-cell development, intestinal physiology, and ultimately T1D development.

Translating this work toward a potential method for T1D prevention in humans required a pilot study in healthy individuals. Hence, the primary aim of this study was to assess the safety and tolerability of *L. johnsonii* N6.2. A secondary mechanistic aim was to characterize the host immune response to *L. johnsonii* N6.2 consumption, specifically the impact of this bacterium on circulating immunoglobulin, cytokines, leukocyte subpopulations, and relevant metabolites in the IDO pathway in healthy adults.

## Materials and Methods

### Subjects

Forty-two healthy adults (female = 30, male = 12; mean age ± SD = 23.2 ± 5.5 years) participated in the study. Participants were recruited from the community and the University of Florida campus in Gainesville, FL, USA in accordance with an Institutional Review Board (IRB) approved study at the University of Florida. Exclusion criteria included gastrointestinal disease (gastric ulcers, Crohn’s, ulcerative colitis, etc.), chronic disease such as diabetes, kidney disease, and heart disease; current or past treatment for immune-compromising diseases or conditions; currently working or living with an immunocompromised person; currently taking medications for constipation, diarrhea, or a psychological disorder (depression, anxiety, insomnia, etc.); antibiotics within the past 4 weeks prior to randomization; currently taking a probiotic supplement and unwilling to discontinue a minimum of 2 weeks prior to the study start; current smoker; pregnant or lactating or a female who plans to become pregnant in the next 6 months; and a known allergy to milk. Inclusion criteria included men and women 18–50 years of age and approval to participate following screening blood work and physical examination by the advising physician.

### Experimental Design

In a double-blinded study, healthy volunteers were randomly assigned to one of two treatments, *L. johnsonii* N6.2 at 5 × 10^8^ colony-forming units (CFU) per capsule or placebo (skim milk) capsule for 8 weeks in a parallel design. Prior to treatment, there was a 1-week pre-baseline period, and treatment was followed by a 4-week washout period. One week prior to randomization, consented participants underwent a physical examination and were screened *via* a comprehensive metabolic panel (CMP), and females received a pregnancy test. During pre-baseline, intervention and washout periods, participants completed a daily online questionnaire, reporting on: study supplement intake, hours of sleep, bowel movement frequency, gastrointestinal symptoms, general wellness, and medication use. In addition, participants completed the gastrointestinal symptom rating scale (GSRS), and quality of life was assessed with the quality of life questionnaire, SF-36v2^®^ on a weekly basis. At the randomization appointment, height, weight, vitals (blood pressure, heart rate), and demographic information were obtained. CMP and complete blood count were assessed at baseline, during weeks 2, 4, and 8 of the study intervention, and during washout. An additional pregnancy test was given during week 4 of the intervention phase. At a final appointment, participants returned any unconsumed supplements.

### *L. johnsonii* N6.2 Culture and Capsules Elaboration

*Lactobacillus johnsonii* N6.2 was grown in modified MRS medium (FGM-LJ2). The media contained peptone 10 g, meat powder 10 g, yeast peptone 5 g, table sugar 20 g, K_2_HPO_4_ 2 g, sodium acetate 5 g, ammonium citrate tribasic 2 g, MgSO_4_⋅7H_2_O 0.2 g, MnSO_4_⋅H_2_O 0.05 g, tween 80 1 g; final volume of 1 L with DI water. *L. johnsonii* N6.2 was incubated at 37°C for 16 h under microaerophilic conditions. Cells were pelleted by centrifugation at 6,000 *g* for 20 min at 4°C and washed twice with BAM R61 0.02 M phosphate buffer pH 7.3 (Bacteriological Analytical Manual, 8th Edition, Revision A, 1998). The cell pellet was resuspended in sterile reconstituted food grade skim milk at 100 g/L (Real Food, IL, USA), transferred to sterile bags (Whirl-Pak, USA), and frozen at −80°C for at least 2 h. The frozen samples were freeze dried (LabConco FreeZone, LabConco Corp., MO, USA) for 48 h. The dried powder was saved at 4°C until capsule filling. Acid resistant capsules (AR Caps, Size #1, CapsCanada, Pompano Beach, FL, USA) were filled using a sterilized Profiller (Torpac^®^, NJ, USA) with the freeze-dried preparation of *L. johnsonii* N6.2 in skim milk (Real Food, IL, USA). Lyophilized skim milk in identical capsules was used as the placebo. The study capsules were provided in bottles labeled with treatment codes by a study collaborator who did not have contact with study participants.

### Stool Sample Collection and Transit Survival of *L. johnsonii* N6.2

Single stools were collected using a commode specimen collection system (Fisher Scientific, Pittsburgh, PA, USA) during the last 2 days of pre-baseline, during weeks 2, 4, and 8 of the intervention, and during washout. Participants were instructed to place the stool containers on ice immediately after defecation and deliver samples to study personnel within 4 h of defecation. Samples were homogenized and fractionated on sterile vials (approximately 1.0 g/vial) and saved at −80°C until use. Fresh samples (approximately 1 g) were immediately diluted (1/10 w/v) in phosphate buffer solution (pH: 7.4), and serial dilutions were made and plated on MRS agar media (pH; 5.5 ± 0.1). Plates were incubated for 48 h under microanaerobic conditions. Values were referred as CFU per wet gram stool (CFU/g). The identity of *L. johnsonii* N6.2 was confirmed by PCR amplification of the strain-specific gene T285_00345 gene (26).

### Blood Sample Collection and CMP

From fasting blood samples, serum (Red top Tube, BD, USA) and plasma (EDTA Purple top Tube, BD, USA) were collected. The CMP was obtained from serum samples evaluating glycemia (glucose level), kidney function (creatinine and urea level), and liver function (aspartate aminotransferase, alanine aminotransferase, alkaline phosphatase, and bilirubin level). The analysis was performed by Vista Clinical Diagnostics, Clermont, FL, USA. Additionally, plasma and serum samples were aliquoted, flash frozen in liquid nitrogen, and stored at −80°C for further assays. ELISA assays were used to quantify serum insulin levels (eBioscience, CA, USA) and C-reactive protein (CRP) (Cayman Chemical, MI, USA).

### IDO Activity: Tryptophan Metabolites Pathway

Quantification of tryptophan catabolites and other metabolites in blood plasma samples (i.e., tryptophan, kynurenine, kynurenic acid, xanthurenic acid, serotonin, and anthranilic acid) were performed using global high-performance liquid chromatography and mass spectrometry (LC-HRMS/HRMS) at the Southeast Center for Integrated Metabolomics of the University of Florida.

### Cytokine Determinations

The following ELISA kits were used: IL-2, IL-6, and TNF-α from eBioscience (CA, USA); IFN-γ and IFN-α from Abcam (MA, USA); IL-2SsRα from BD (NJ, USA) following the manufacturers’ instructions.

### Flow Cytometry

Direct immunofluorescence surface staining of whole blood samples with six antibody panels was performed to provide a detailed assessment of immune cell subsets. Five of the panels and gating strategies are emulated from Maecker (27) and consist of a B cell subset panel (CD3, CD19, CD20, CD24, CD27, CD38, and IgD), innate cell panel (CD3, CD11c, CD14, CD16, CD19, CD20, CD56, CD123, and HLA-DR), T cell naïve and memory panel (CD3, CD4, CD8, CD38, CD45RA, CD197, and HLA-DR), T cell effector subset panel (CD3, CD4, CD38, CD45RO, CD183, CD196, and HLA-DR), and a Treg panel (CD3, CD4, CD25, CD45RO, CD127, CD194, and HLA-DR). A sixth follicular helper T (Tfh) panel (CD3, CD4, CD45RA, CD183, CD196, CD197, and CD279) was designed to assess precursor Tfh (28) and memory Tfh (29). Antibodies against the following antigens were used: CD3 (SK7), CD8 (SK1), CD19 (HIB19), CD20 (2H7), and CD45RO (UCHL1) from BD Biosciences, HLA-DR (LN243), IgD (IA6-2), CD3 (UCHT1), CD4 (RPA-T4), CD11c (Bu15), CD14 (M5E2), CD16 (3G8), CD24 (ML5), CD25 (BC96), CD27 (O323), CD38 (HB7), CD45RA (HI100), CD56 (HCD56), CD123 (6H6), CD127 (A019D5), CD183 (G025H7), CXCR3 (G025H7), CD185 (J252D4), CD194 (L291H4), CD196 (G034E3), and CD197 (G043H7) from BioLegend (USA), and CD279 (eBioJ105) from eBiosciences (USA). Whole blood (200 μL/stain) was incubated for 30 min at room temperature and protected from light. Afterward, 2 mL of Fix/lyse 1× (eBioscience, USA) was added and incubated at room temperature for 5 min. Successive washing/centrifugation (5 min, 450 g) steps were performed until hemolysis color completely faded. The samples were acquired on a BD Fortessa cytometer, and data were analyzed by FlowJo software.

### Extraction of Fecal Microbiota

DNA was extracted from fecal samples and preserved at −80°C using the PowerFecal^®^ DNA isolation kit (MoBio Lab, Inc., USA) with the following modification. 250 mg of fecal sample were homogenized in 750 µL of bead solution and 100 µL of Protease from *Streptomyces griseus* 20 mg/mL (Sigma-Aldrich, Steinheim, Germany) were added. The mixture was incubated at 37°C for 15 min and samples were processed according to the manufacturers’ protocol. In the elution step, the DNA was collected in 70 µL of water and quantified. The DNA concentration was standardized to 1 ng/µL before the amplification of the V4 region using primers for paired-end sequencing on the Illumina MiSeq platform as described earlier (30).

### Microbiota Analysis

Clustering of operational taxonomic units (OTUs) at 97% similarity was performed with the subsampled open-reference OTU picking method (31), with no removal of singletons. The Greengenes reference dataset version 13.8 (32) was used as the reference for OTU picking and for taxonomy assignment with uclust (33). OTUs identified as mitochondrial DNA or as chloroplasts were removed from further analyses. Parsed raw sequencing reads are publicly available through NCBI’s Sequence Read Archive under the BioProject accession number PRJNA378749.

### Statistics

Unless otherwise noted, statistical analysis was performed using JMP Pro software (SAS Institute, Cary, NC, USA). Multivariate analysis was performed by two-way analysis of variance (ANOVA) with a *post hoc* Tukey’s honestly significant difference test. Bivariate analysis was performed using Student’s *t*-tests. Numerical data are summarized as mean ± SE. Significance was defined as *p* < 0.05.

#### Analyses of Surveys

For each of the response variables, a repeated measures analysis was performed by fitting a linear mixed model that considered the repeated nature of the data. The fitted model had the following form: *y* = μ + gender + supp + group(supp) + *e* where μ is the overall mean, gender is the gender effect, supp is a diet supplementary effect, group (supp) corresponds to the combination of measurement week within a diet supplement effect, and *e* corresponds to an error term, where measurements from the same individual were correlated using an unstructured error with a different correlation for each pair of time points and a different error variance for each time point. The models were fitted using SAS v. 9.4 with the procedure MIXED and degrees of freedom were adjusted using the Kenward–Rogers correction. Comparisons of means for the diet supplement levels at a given week were obtaining constructing specific contrasts, and for all tests a significance level of 5% was considered.

#### Analyses of Immune Cells

For each of the response variables, a repeated measures analysis was performed by fitting a linear mixed model that considered the repeated nature of the data. The fitted model had the following form: *y* = μ + β*x0 + gender + supp + group + time + supp*time + group*time + supp*group + supp*group*time + *e* where μ is the overall mean, β*x0 is the regression coefficient associated with the covariate for initial measurement β*x0, gender is the gender effect, supp is a diet supplementary effect, group is the group effect, and time is the time of measurement. The other terms are the two- and three-way interactions. Also, *e* corresponds to an error term, where measurements from the same individual were correlated using an unstructured error with a different correlation for each pair of time points and a different error variance for each time point. The models were fitted using SAS v. 9.4 with the procedure MIXED and degrees of freedom were adjusted using the Kenward–Rogers correction. Comparisons of means for a given model term were obtained with the least significance difference, and for all tests a significance level of 5% was considered.

#### Analysis of Microbiota

Community structure was analyzed in R with phyloseq (34) and plotted with ggplot2 (35). Analysis of similarities (ANOSIM) and Permutational Multivariate Analysis of Variance (PERMANOVA) were performed in R using VEGAN v2.0-8 (36). Differences in taxonomic profiles were analyzed by Welch’s *t*-test (for two groups) or by ANOVA (for multiple groups) with Tukey–Kramer *post hoc* tests with STAMP (37).

### Study Approval

The study was approved by the IRB (# 201400370) at the University of Florida and conducted according to guidelines established by the Declaration of Helsinki. Participants were informed of the aims, requirements, and risk/benefits of the study, and written informed consent indicating their full knowledge of the study protocol was received from participants prior to study enrollment. In addition, an Investigational New Drug (IND#016829) has been filed with the Food and Drug Administration of the United States of America.

## Results

### The Administration of *L. johnsonii* N6.2 Decreases Indigestion, Abdominal Pain, and Cephalic Syndrome Scales

**Figure 1:**
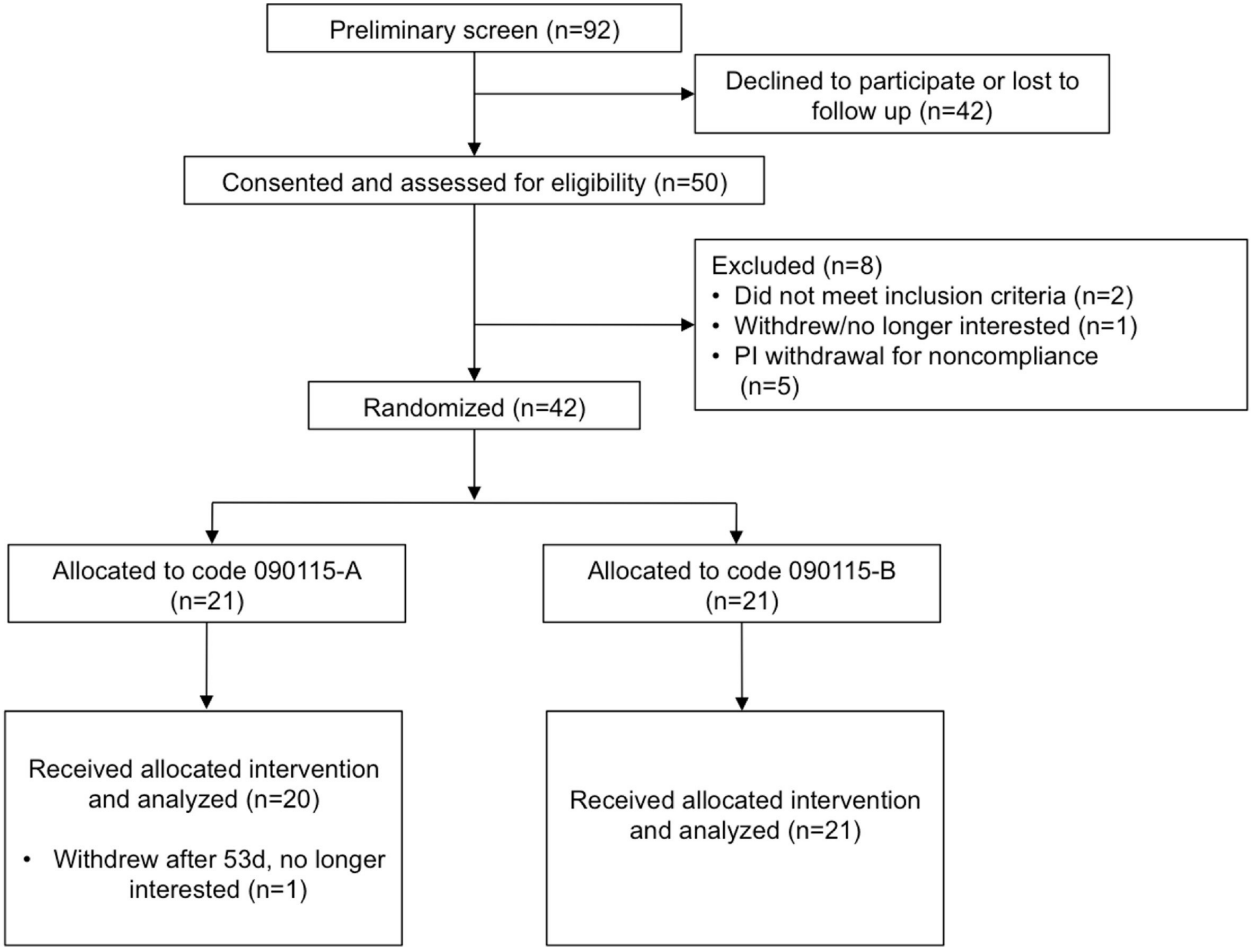
Study flow diagram to illustrate the number of subjects that were screened, consented, and randomized. Code 090115-A was used for the placebo, whereas Code 090115-B was used for *Lactobacillus johnsonii* N6.2.

Of the 92 individuals initially screened and assessed for eligibility, 50 were consented, and 42 randomized to the treatment groups (Figure 1) in a double-blind parallel study design. Table 1 summarizes the characteristics and compliance of the subjects that participated in the study.

**Table 1:** Characteristics of the participants and compliance.

Measure	Placebo (*n* = 21)	Ljo^a^ (*n* = 21)
Gender (M/F), *n*	7/14	5/16
Age, years median (range)	21 (18–48)	23 (18–36)
Race/ethnicity, *n* (%)		
Asian	2 (10%)	4 (19%)
African-American	1 (5%)	1 (5%)
Hispanic	3 (14%)	4 (19%)
White	18 (86%)	12 (57%)
Other	0	3 (14%)^b^
BMI, mean (SD)	23.6 ± 4.7	23.6 ± 4.2
Blood pressure (mean mm Hg)	116/75	119/74
Compliance (%)		
Supplement protocol	88.7 ± 10.0	92.9 ± 8.3
Questionnaires protocol	86.6 ± 14.4	90.7 ± 9.7

*^a^Lactobacillus johnsonii N6.2*.

*^b^Participants who classified themselves as other included n = 2 Hawaiian and n = 1 Unknown*.

The analysis of the hemogram and CMP data showed no statistically significant differences between the *L. johnsonii* N6.2 and placebo groups (Table S1 in Supplementary Material). The only parameter that showed statistical significance after 8 weeks of treatment was total bilirubin values (placebo = 0.54 ± 0.05 versus *L. johnsonii* N6.2 = 0.70 ± 0.05, *p* < 0.05); however, both groups were within the reference range of 0.2–1.9 mg/dL (38). These differences were not observed after the washout period (12 weeks). No statistically significant differences were observed in either the control or treatment cohorts with regards to kidney and liver function (Table S1 in Supplementary Material). As expected, no alterations in the circulating levels of insulin and CRP were observed in the *L. johnsonii* N6.2 group at 8 weeks or 12 weeks compared to placebo. Based on these results, the consumption of *L. johnsonii* N6.2 was well tolerated without apparent risks or deviations from the reference ranges for standard clinical assessments.

We next assessed general digestive health over the course of the trial. During pre-baseline, intervention, and washout periods, participants completed a daily online questionnaire reporting study supplement intake, hours of the sleep, bowel movement frequency, gastrointestinal symptoms, general wellness, and medication use. Of the five domains of the weekly GSRS questionnaire, indigestion (*p* < 0.05) and abdominal pain (*p* < 0.05) were significantly lower among *L. johnsonii* N6.2 treated subjects during treatment weeks 2 to 8, and during washout weeks 1–4 compared to placebo (Table 2). Syndrome scores from the daily questionnaire indicated that cephalic syndrome, including the symptoms of headache and dizziness, was significantly lower (*p* < 0.05) for *L. johnsonii* N6.2 versus placebo for treatment weeks 2 and 4–8. The gastrointestinal distress syndrome was lower (*p* < 0.05) for *L. johnsonii* N6.2 versus placebo during most treatment weeks and neared significance at baseline (*p* = 0.05). Interestingly, the probiotic group showed a significant decrease (*p* < 0.05) in the epidermal syndrome at the end of the washout period (Table 3). Stomach ache or pain as an individual symptom was significantly lower in the group receiving *L. johnsonii* N6.2 during treatment weeks 1–8 compared to the placebo group, with a similar trend at baseline (*p* = 0.06) (Table S2 in Supplementary Material). Bloating as an individual symptom was lower (*p* < 0.01) in *L. johnsonii* N6.2 versus placebo at baseline and during most treatment and washout weeks (Table S2 in Supplementary Material). Administration of *L. johnsonii* N6.2 also resulted in lower individual daily symptoms of cramping, abdominal noises, and headache for most treatment weeks. Furthermore, a significant decrease in the anxiety symptom after the washout period in the probiotic group was observed (Table S2 in Supplementary Material). The anxiety changes may have affected the psychology syndromes where a trend to decrease (*p* < 0.1) during the washout was observed (Table 3).

**Table 2:** Gastrointestinal symptom rating scale scores.

Period	Abdominal pain^a^		Reflux^b^		Diarrhea^c^		Indigestion^d^		Constipation^e^	
	Placebo	Ljo	Placebo	Ljo	Placebo	Ljo	Placebo	Ljo	Placebo	Ljo
Baseline	1.6 ± 0.1	1.3 ± 01	1.2 ± 0.1	1.1 ± 0.1	1.4 ± 0.2	1.3 ± 0.1	1.9 ± 0.1	1.4 ± 0.1*	1.4 ± 0.1	1.3 ± 0.1
Week 1	1.7 ± 0.1	1.2 ± 0.1*	1.1 ± 0.1	1.0 ± 0.1	1.3 ± 0.1	1.4 ± 0.1	1.7 ± 0.1	1.4 ± 0.1*	1.3 ± 0.1	1.1 ± 0.1
Week 2	1.7 ± 0.1	1.1 ± 0.1***	1.2 ± 0.1	1.1 ± 0.1	1.5 ± 0.2	1.3 ± 0.2	1.8 ± 0.1	1.3 ± 0.1***	1.5 ± 0.2	1.2 ± 0.2
Week 3	1.4 ± 0.1	1.1 ± 0.1*	1.3 ± 0.1	1.1 ± 0.1	1.4 ± 0.1	1.3 ± 0.1	1.7 ± 0.1	1.3 ± 0.1*	1.4 ± 0.1	1.2 ± 0.1
Week 4	1.5 ± 0.1	1.1 ± 0.1*	1.2 ± 0.1	1.1 ± 0.1	1.5 ± 0.2	1.4 ± 0/1	2.0 ± 0.1	1.3 ± 0.1**	1.5 ± 0.2	1.2 ± 0.1
Week 5	1.5 ± 0.1	1.1 ± 0.1**	1.2 ± 0.1	1.0 ± 0.1	1.4 ± 0.1	1.2 ± 0.1	1.8 ± 0.2	1.3 ± 0.1*	1.3 ± 0.1	1.2 ± 0.1
Week 6	1.4 ± 0.1	1.1 ± 0.1*	1.3 ± 0.2	1.2 ± 0.2	1.5 ± 0.2	1.2 ± 0.2	1.6 ± 0.1	1.3 ± 0.1*	1.3 ± 0.1	1.2 ± 0.1
Week 7	1.6 ± 0.1	1.0 ± 0.1**	1.2 ± 0.1	1.0 ± 0.1*	1.2 ± 0.1	1.1 ± 0.1	1.7 ± 0.1	1.2 ± 0.1**	1.4 ± 0.1	1.1 ± 0.1*
Week 8	1.6 ± 0.1	1.1 ± 0.1**	1.2 ± 0.1	1.0 ± 0.1	1.4 ± 0.1	1.1 ± 0.1	1.7 ± 0.1	1.2 ± 0.1**	1.6 ± 0.1	1.1 ± 0.1*
Washout 1	1.5 ± 0.1	1.0 ± 0.1**	1.2 ± 0.1	1.0 ± 0.1*	1.5 ± 0.1	1.1 ± 0.1*	1.8 ± 0.1	1.2 ± 0.1**	1.4 ± 0.1	1.1 ± 0.1*
Washout 2	1.4 ± 0.1	1.0 ± 0.1***	1.1 ± 0.1	1.0 ± 0.1	1.3 ± 0.2	1.2 ± 0.2	1.7 ± 0.1	1.3 ± 0.1*	1.5 ± 0.1	1.1 ± 0.1*
Washout 3	1.7 ± 0.1	1.1 ± 0.1***	1.3 ± 0.1	1.1 ± 0.1	1.4 ± 0.1	1.2 ± 0.1	1.9 ± 0.1	1.3 ± 0.1***	1.4 ± 0.1	1.1 ± 0.1*
Washout 4	1.5 ± 0.1	1.2 ± 0.1	1.3 ± 0.1	1.2 ± 0.1	1.4 ± 0.1	1.1 ± 0.1*	1.8 ± 0.1	1.2 ± 0.1***	1.3 ± 0.1	1.1 ± 0.1

*^a^Abdominal pain syndrome includes abdominal pain, hunger pains, and nausea symptoms*.

*^b^Reflux syndrome includes heartburn and acid regurgitation symptoms*.

*^c^Indigestion syndrome includes stomach rumbling, bloating, burping, and increased flatus symptoms*.

*^d^Constipation syndrome includes constipation, hard stools, and feeling of incomplete evacuation symptoms*.

*^e^Diarrhea syndrome includes diarrhea, loose stools, and urgent need for defecation symptoms*.

*Ljo correspond to Lactobacillus johnsonii N6.2*.

*Data presented as least squares mean ± SEM*.

*p < 0.05.

**p < 0.01.

***p < 0.001.

**Table 3:** Daily questionnaire syndrome scores.

Period	GI distress^a^		Epidermal^b^		Cephalic^c^		Ear–nose–throat^d^		Psychological^e^		Emetic^f^	
	Placebo	Ljo	Placebo	Ljo	Placebo	Ljo	Placebo	Ljo	Placebo	Ljo	Placebo	Ljo
Baseline	3.1 ± 0.5	1.6 ± 0.5	0.3 ± 0.2	0.1 ± 0.2	0.4 ± 0.1	0.5 ± 0.1	0.7 ± 0.2	0.6 ± 0.2	1.7 ± 0.6	2.6 ± 0.6	0.07 ± 0.06	0.13 ± 0.06
Week 1	2.2 ± 0.3	1.1 ± 0.3*	0.5 ± 0.2	0.01 ± 0.2	0.4 ± 0.1	0.4 ± 0.1	1.2 ± 0.4	0.7 ± 0.4	1.2 ± 0.5	2.0 ± 0.5	0.06 ± 0.03	0.02 ± 0.03
Week 2	1.7 ± 0.3	1.1 ± 0.3	0.6 ± 0.2	0.01 ± 0.2*	0.9 ± 0.2	0.2 ± 0.2**	1.3 ± 0.3	0.4 ± 0.3*	2.1 ± 0.6	1.4 ± 0.6	0.09 ± 0.06	0.01 ± 0.06
Week 3	2.0 ± 0.4	0.8 ± 0.4*	0.5 ± 0.2	0.07 ± 0.2	0.4 ± 0.1	0.3 ± 0.1	1.1 ± 0.4	0.8 ± 0.4	1.7 ± 0.4	0.9 ± 0.4	0.08 ± 0.07	0.13 ± 0.07
Week 4	2.2 ± 0.3	1.0 ± 0.3**	0.4 ± 0.2	0.07 ± 0.2	0.5 ± 0.1	0.05 ± 0.1**	1.1 ± 0.3	0.3 ± 0.3*	1.7 ± 0.6	1.6 ± 0.6	0.10 ± 0.05	0.01 ± 0.05
Week 5	1.9 ± 0.3	1.1 ± 0.3*	0.4 ± 0.2	0.03 ± 0.2	0.6 ± 0.1	0.2 ± 0.1*	1.2 ± 0.3	0.4 ± 0.3	1.9 ± 0.5	1.2 ± 0.5	0.10 ± 0.05	0.02 ± 0.05
Week 6	1.5 ± 0.2	0.9 ± 0.2	0.3 ± 0.2	0.02 ± 0.2	0.6 ± 0.1	0.02 ± 0.1**	1.0 ± 0.3	0.3 ± 0.3	1.8 ± 0.5	1.0 ± 0.5	0.08 ± 0.03	0.02 ± 0.03*
Week 7	2.0 ± 0.2	0.8 ± 0.2**	0.3 ± 0.1	0.01 ± 0.1	0.6 ± 0.1	0.04 ± 0.1***	0.8 ± 0.2	0.2 ± 0.2*	1.7 ± 0.5	1.0 ± 0.5	0.19 ± 0.07	0.01 ± 0.07
Week 8	1.6 ± 0.3	0.7 ± 0.3*	0.4 ± 0.2	0.02 ± 0.2	0.3 ± 0.1	0.04 ± 0.1*	0.5 ± 0.2	0.1 ± 0.2	1.8 ± 0.4	0.8 ± 0.4	0.04 ± 0.03	0.02 ± 0.03
Washout 1	2.1 ± 0.3	0.8 ± 0.3**	0.2 ± 0.1	0.01 ± 0.1	0.3 ± 0.1	0.03 ± 0.1	0.8 ± 0.3	0.2 ± 0.3	1.8 ± 0.4	0.8 ± 0.4	0.06 ± 0.04	0.02 ± 0.04
Washout 2	2.2 ± 0.4	0.7 ± 0.4**	0.2 ± 0.1	0.01 ± 0.1*	0.6 ± 0.1	0.04 ± 0.1***	0.6 ± 0.2	0.3 ± 0.2	1.6 ± 0.4	0.8 ± 0.4	0.11 ± 0.03	0.02 ± 0.03*
Washout 3	2.6 ± 0.5	1.0 ± 0.5*	0.3 ± 0.1	0.07 ± 0.1*	0.4 ± 0.1	0.1 ± 0.1	0.7 ± 0.2	0.4 ± 0.2	1.9 ± 0.4	1.1 ± 0.4	0.17 ± 0.06	0.06 ± 0.06
Washout 4	1.9 ± 0.4	0.7 ± 0.4*	0.3 ± 0.1	0.07 ± 0.1*	0.4 ± 0.1	0.1 ± 0.1	1.0 ± 0.3	0.4 ± 0.3	2.1 ± 0.5	1.0 ± 0.5	0.08 ± 0.05	0.08 ± 0.05

*^a^Gastrointestinal distress syndrome includes daily symptoms of bloating, flatulence, stomach noises, and abdominal cramps*.

*^b^Epidermal syndrome includes daily symptoms of itching, skin rash, and skin redness/flushing*.

*^c^Cephalic syndrome includes daily symptoms of headache and dizziness*.

*^d^Ear–nose–throat syndrome includes daily symptoms of sore throat, runny eyes, nasal congestion, and blocked ear canal*.

*^e^Psychological syndrome includes daily symptoms of anxiety, depression, and stress*.

*^f^Emetic syndrome includes daily symptoms of nausea and vomiting*.

*Ljo correspond to Lactobacillus johnsonii N6.2*.

*Data presented as least squares mean ± SEM*.

*p < 0.05.

**p < 0.01.

***p < 0.

### *L. johnsonii* N6.2 Survives Human Gastrointestinal Transit

Gastrointestinal transit survival of *L. johnsonii* N6.2 was evaluated by following the total CFU/g of stools counts of lactic acid bacteria (LAB) in fresh fecal samples. Additionally, the presence of *L. johnsonii* N6.2 was confirmed by RT-PCR of the T285_00345 gene.

**Figure 2:**
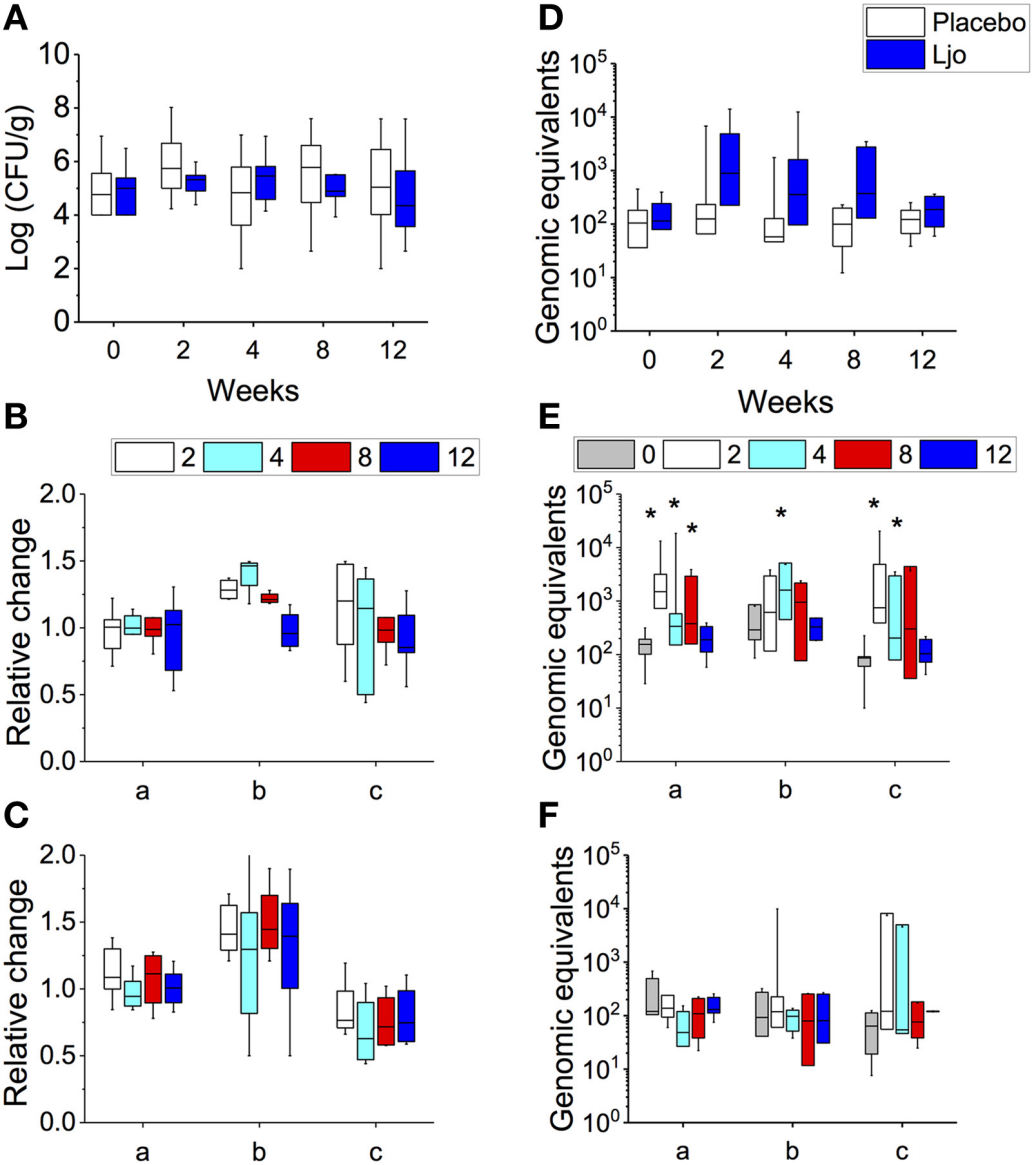
Determination of total lactic acid bacteria (LAB) and *L. johnsonii* N6.2 (Ljo) in stool samples. In placebo and Ljo groups, it was determined: **(A)** total number of LAB (Log CFU/g). Based on the numbers of LAB obtained, three subgroups were defined: (a) high LAB, (b) low to high LAB, and (c) low LAB. **(B)** Relative change in LAB for Ljo. **(C)** Relative change in LAB for placebo. **(D)** The presence of Ljo was confirmed by performing qRT-PCR of the T285_00345 gene and expressed as genomic equivalents. These data were further stratified based on the determination of total LAB numbers for the Ljo **(E)** and placebo **(F)** treatment groups. * indicates statistical differences (*p* < 0.05) between the groups and time points shown in panels **(E,F)** using analysis of variance. Comparison of the treatment combinations was performed by least significance difference with a significance level of 5%.

Overall, a large variability in the amount of the total CFU of LABs was observed between subjects (from 10^2^ to 10^8^ CFU/g stools) at time 0, and no significant changes were observed over time either in the placebo or in the *L. johnsonii* N6.2 treatment group (Figure 2A). However, it was possible to observe three groups of subjects within each treatment: (a) subjects with high counts of LAB throughout the study (>10^5^ CFU/g), (b) a group that at time 0 showed low concentrations of LAB that increased over time (from 10^4^ to 10^8^ CFU/g), and (c) subjects with low counts of LAB throughout the study (<10^5^ CFU/g). To quantify the variation of the LAB population, the log CFU/g values for time 0 was subtracted at each time point and expressed as relative change (log CFU/g_at each time point_/log CFU/g_at time 0_). For subjects with consistently high or low LAB populations (groups a and c), the relative fold change in LAB was 1.0 ± 0.11 and 0.8 ± 0.02, respectively, in the probiotic group (Figure 2B). Similar results were obtained in the placebo group (Figure 2C). As expected, subjects in group b that received *L. johnsonii* N6.2 capsules displayed the highest relative change in LAB counts (Figure 2B). After the washout period, the LAB counts appeared to return to baseline levels (Figures 2B,C). These results suggest that *L. johnsonii* N6.2 survived the transit through the gastrointestinal system and potentially may not colonize the gut.

To verify this hypothesis, the presence of *L. johnsonii* N6.2 was confirmed using specific RT-PCR of the T285_00345 gene and expressed as genomic equivalents/100 ng of DNA (Figure 2D). This gene was found in the genome of *L. johnsonii* N6.2 but not in others in the NCBI database. It was found that the T285_00345 gene gave background amplification on the placebo-treated group (Figure 2F), while a significant increase in genomic equivalents over time (*p* < 0.05) were observed in the *L. johnsonii* N6.2 group (Figure 2E). Interestingly, the presence of *L. johnsonii* N6.2 was confirmed in all the subgroups (a, b, and c) independently of the total LAB counts (see Figures 2E,F). However, after washout, the genomic equivalents in *L. johnsonii* N6.2 group were similar to time 0 or below the detection limit.

### *L. johnsonii* N6.2 Modulates the Concentration of Metabolites in the IDO-Dependent Pathway in Healthy Subjects

We previously reported that the administration of *L. johnsonii* N6.2 to BB-DP rats resulted in decreased expression of IDO and, consequently, changes in the kynurenine:tryptophan (K:T) ratios in peripheral serum (25). Here, the impact of *L. johnsonii* N6.2 on IDO activity was evaluated by quantifying plasma levels of the following metabolic intermediates in the tryptophan pathway: tryptophan, kynurenine, serotonin, xanthurenic acid, anthranilic acid, and kynurenic acid. Samples taken at different time points (0, 8, and 12 weeks) were quantified using liquid chromatography-mass spectrometry (LC-HRMS/HRMS) (Table S3 in Supplementary Material). Based on our findings in rodent studies, it was expected that a decrease in IDO activity or expression would increase the concentration of tryptophan, while decreasing the concentrations of kynurenine, xanthurenic acid, anthranilic acid, and kynurenic acid associated also with a possible increase in serotonin levels (25). For each of the metabolites, we observed no significant differences between the treatment groups during the treatment period (Table S3 in Supplementary Material). Similarly, the K:T ratio was not affected, being similar for both groups during treatment with placebo or *L. johnsonii* N6.2 (Table S3 in Supplementary Material).

**Figure 3:**
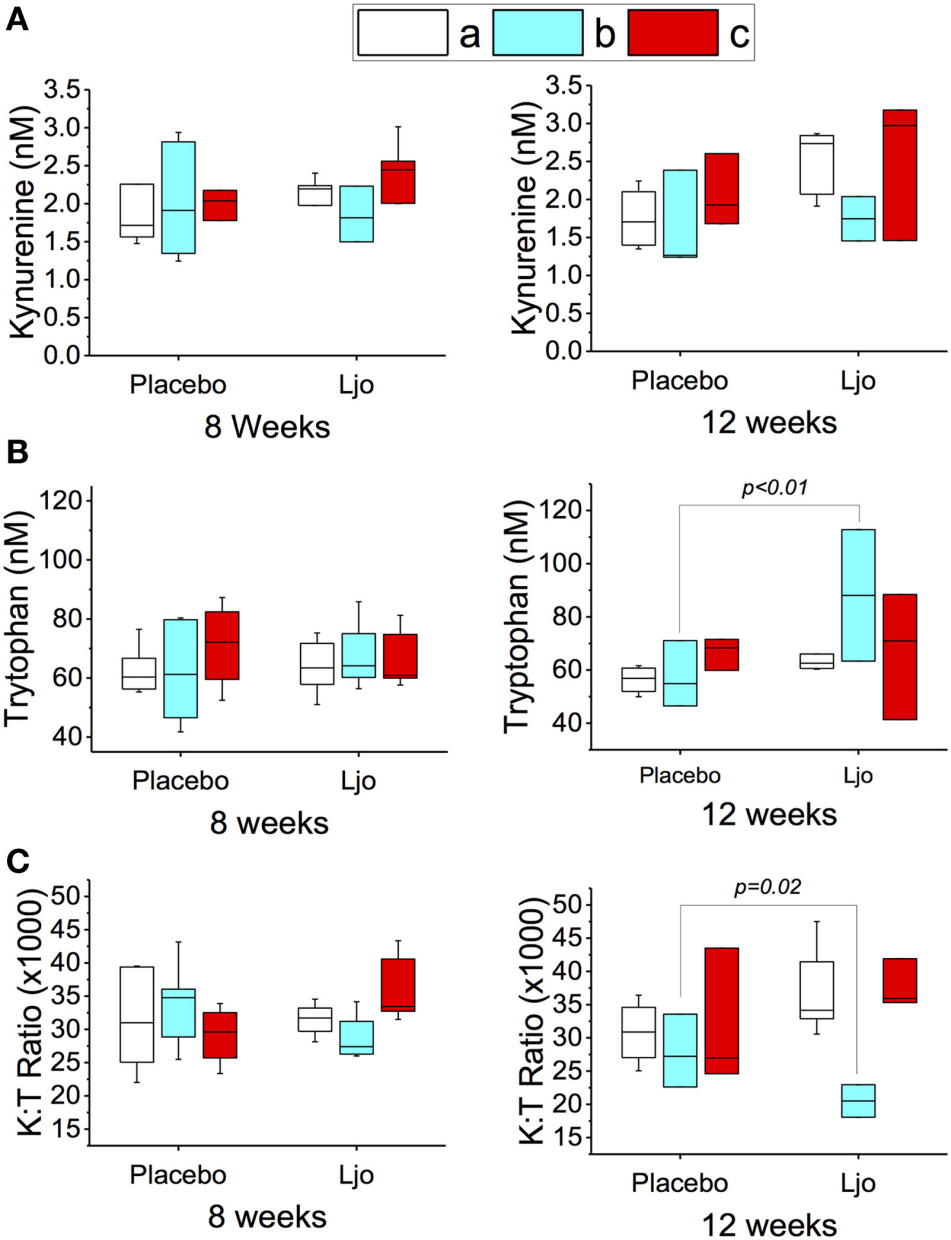
Peripheral tryptophan and kynurenine concentration in plasma of healthy subjects. The concentrations of kynurenine **(A)** and tryptophan **(B)** were determined by LC-HRMS/HRMS after 8 or 12 weeks in the placebo or *L. johnsonii* N6.2 (Ljo) treatment groups. Panel **(C)** is shown the kynurenine:tryptophan (K:T) ratio. The concentration of the metabolites shown has been normalized to the concentration found at time 0 for each subject. The results obtained were further stratified based on the number of LAB present as described in the Section “Results.” (a) High LAB; (b) low to high LAB, and (c) low LAB.

Similar statistical analyses of the metabolic intermediates were also conducted considering the LAB counts (groups a, b, or c as described earlier). After 8 and 12 weeks, the kynurenine values did not change significantly (*p* > 0.1) in the different subgroups (Figure 3A). After 8 weeks (last day of treatment), a slight increase in the tryptophan concentration was observed, which correlated with a decrease in the K:T ratio in the *L. johnsonii* N6.2 versus placebo-treated group b subjects (low to high LAB counts), although statistical significance was not reached (*p* = 0.17 and *p* = 0.13, respectively) (Figures 3B,C). Interestingly, at 12 weeks (after 4 weeks of washout), the changes in tryptophan and K:T ratio reached statistical significance with *p* < 0.01 and *p* < 0.05, respectively (Figures 3B,C). These results suggest that the effects of *L. johnsonii* N6.2 supplementation may take longer than 8 weeks to be quantified.

The fact that the expected modulation of the tryptophan pathway was only observed in one group of subjects (low to high LAB counts), suggests that the effects of *L. johnsonii* N6.2 supplementation may require an intestinal environment that is permissive to microbe colonization over time. These results indicate that the counts of LAB during baseline may also be used as biomarkers to predict responders from non-responders in a heterogeneous population, although this will require confirmation.

### *L. johnsonii* N6.2 Supplementation Alters the Frequency of Immune Subsets in Peripheral Blood

The impact of *L. johnsonii* supplementation on the immune system was evaluated by flow cytometry of PBMCs as described by Maecker (27). The identification of immune cell subsets was performed by eight-color antibody staining at time 0, after 8 weeks of treatment or after the washout period (12 weeks). Six antibody staining panels were used to differentiate the following immune cell subsets of the innate and adaptive arms of the immune system: (i) B cells, (ii) natural killers (NKs), monocytes, and DCs, (iii) naïve and memory T cells, (iv) Tfh cells, (v) differentiated effector T cells (Teff), and (vi) regulatory T cells (Tregs).

#### B Cell Subsets

From the B cell population (CD3^−^CD19^+^), we analyzed the frequencies of transitional (CD27^−^IgD^+^CD24^hi^CD38^hi^), naïve (CD27^−^IgD^+^CD24^lo/−^CD38^lo/−^), non-class switched memory (CD20^hi^CD27^+^IgD^+^), class switched memory (CD20^hi^CD27^+^IgD^−^), or plasmablast (CD20^lo/−^CD38^+^) cells (Figure S1 in Supplementary Material). Analyses of these cell populations either after 8 weeks of treatment or following the washout period indicated that no significant changes were observed upon administration of *L. johnsonii* N6.2 (Table S4 in Supplementary Material).

#### NK, Monocytes, and DC Subsets

**Figure 4:**
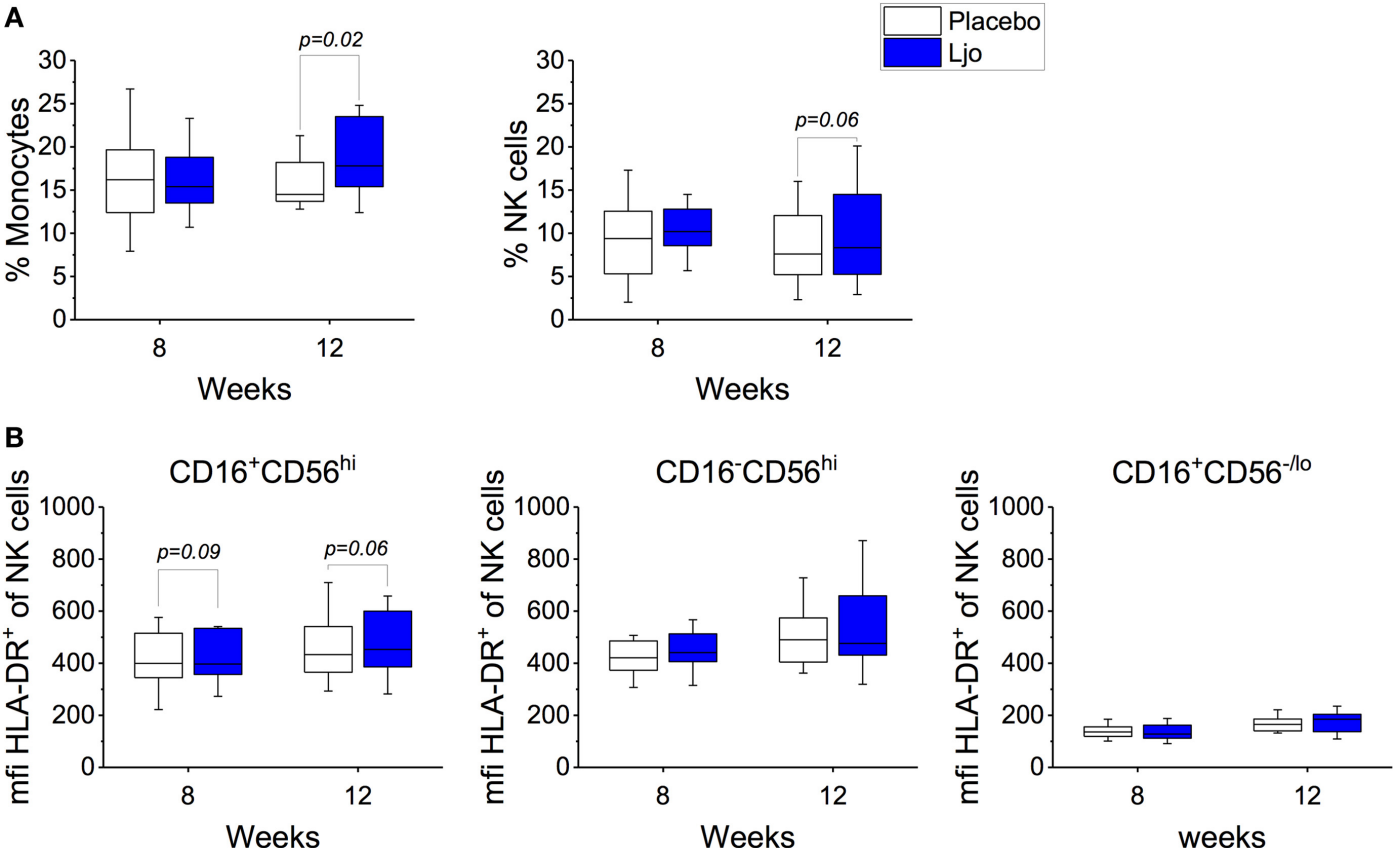
Monocytes and natural killer (NK) cells in healthy subjects. **(A)** Mononuclear cells (CD3^−^CD19^−^) were stained with specific antibodies to define monocytes (CD14^+^) and NK cells (CD14^−^) in the placebo and in the *L. johnsonii* N6.2 (Ljo) groups. **(B)** Expression of HLA-DR (mfi) in different NK cells subset: CD16^−^CD56^hi^, CD16^+^CD56^lo/−^, and CD16^+^CD56^hi^ after 8 weeks of treatment or 12 weeks (4 weeks into the washout). The concentration of cells shown has been normalized to the concentration found at time 0 for each subject.

This staining panel facilitated the discrimination of B and T cell lineage negative cells (CD3^−^CD19^−^CD20^−^) into NK cells (CD56^+^), monocytes (HLA-DR^+^CD14^+^), myeloid DCs (mDCs) (HLA-DR^+^CD14^−^CD16^−^CD11c^+^CD123^−^), and plasmacytoid DCs (pDCs) (HLA-DR^+^CD14^−^CD16^−^CD11c^−^CD123^+^) (Figure S2 and Table S4 in Supplementary Material). It was found that the numbers of mDCs and pDCs neither changed over time nor as a result of the probiotic supplementation. By contrast, the frequencies of monocytes and NK cells were increased as a result of the probiotic treatment reaching statistical significance at 12 weeks (Figure 4A). Specifically, a subset of NK cells (CD16^+^CD56^hi^) showed a trend toward increasing expression of HLA-DR after 8 weeks and after the washout period (*p* < 0.1, Figure 4B). The relative frequency of monocytes was not affected significantly after 8 weeks of treatment (*p* > 0.1); however, at 12 weeks, monocyte frequencies increased significantly among *L. johnsonii* N6.2 treated subjects (*p* < 0.05) (Figure 4A; Table S4 in Supplementary Material).

#### Naïve and Memory T Cell Subsets

**Figure 5:**
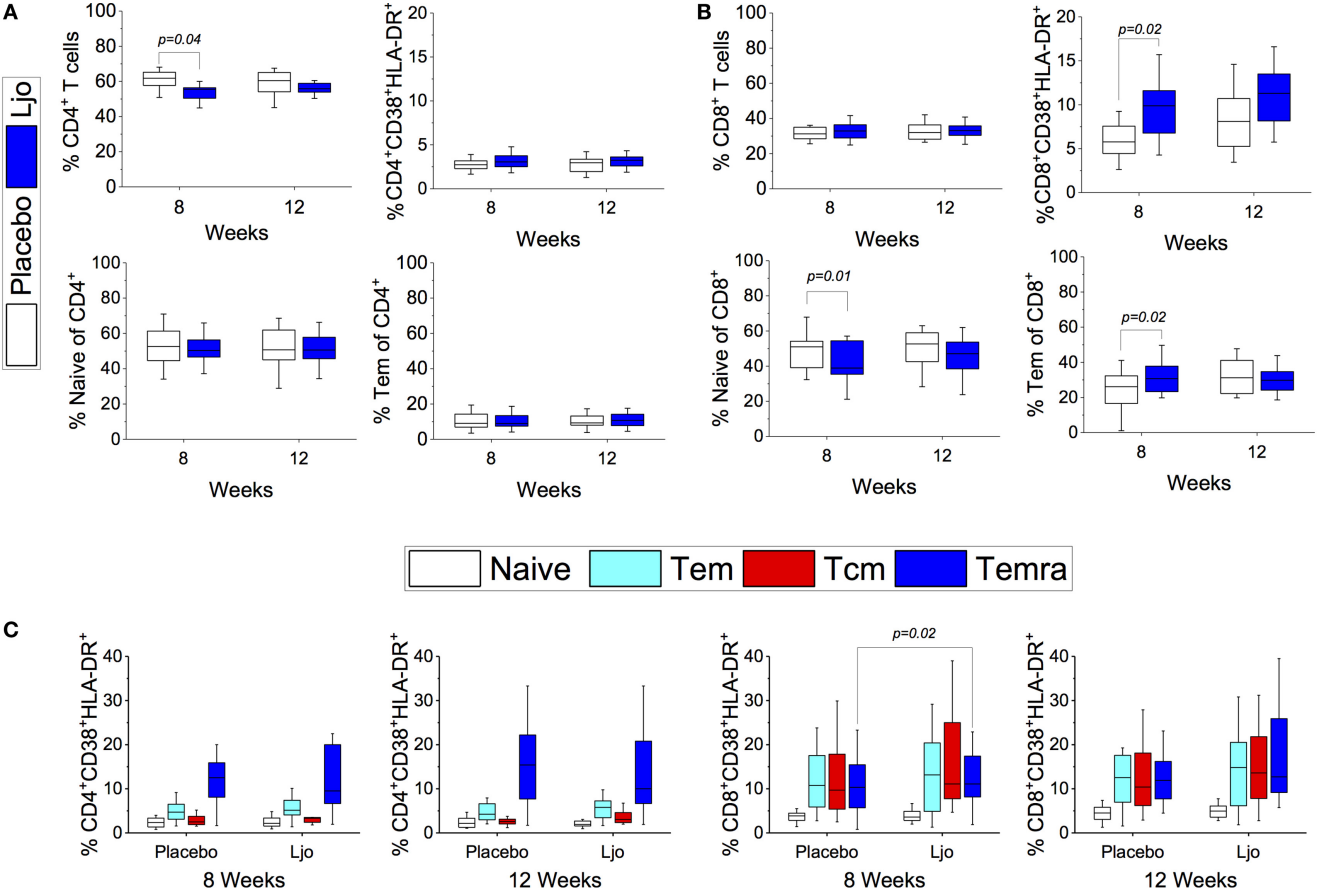
T cell subset. CD4^+^ **(A)** or CD8^+^ **(B)** T cells populations subsets [Naïve, Tem, activated (CD38^+^HLA-DR^+^)] were quantified after 8 or 12 weeks of treatment in the placebo and *L. johnsonii* N6.2 (Ljo) groups. Naïve (CD197^+^CD45RA^+^), Tem (CD197^−^CD45RA^−^), Tcm (CD197^+^CD45RA^−^), and Temra (CD197^−^CD45RA^+^) by labeling with specific antibodies **(C)**. The concentration of cells shown has been normalized to the concentration found at time 0 for each subject.

**Figure 6:**
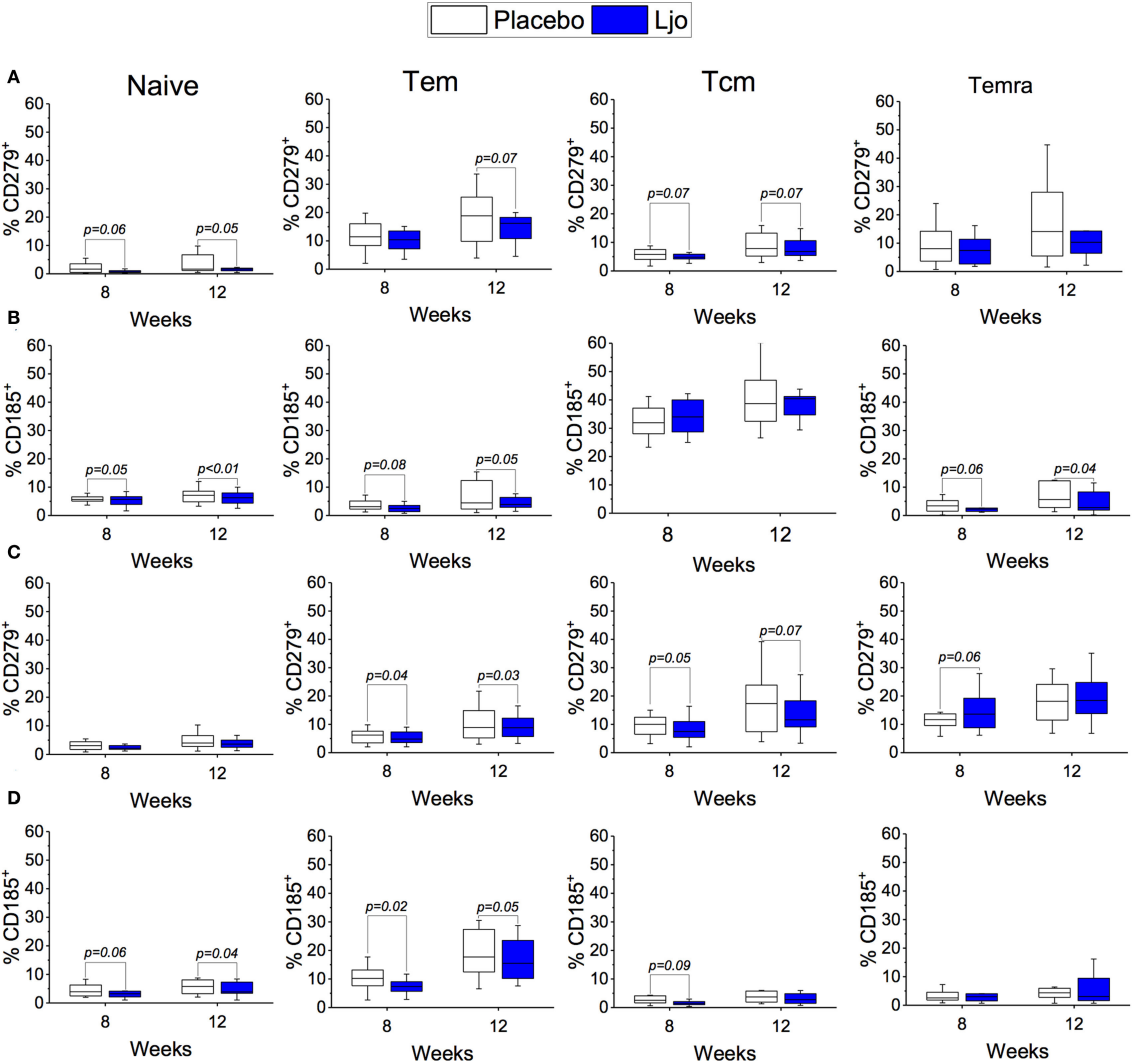
Expression of CD185^+^ and CD279^+^ on T cells subset. Expression of CD279^+^ **(A)** or CD185^+^ **(B)** on Naïve, Tem, Tcm, and Temra CD4^+^ T cells. Expression of CD279^+^ **(C)** or CD185^+^ **(D)** on Naïve, Tem, Tcm, and Temra CD8^+^ T cells. The number of cells in each population was evaluated for the placebo (white bars) and the *L. johnsonii* N6.2 (Ljo, blue bars) group at 8 and 12 weeks. The concentration of cells shown has been normalized to the concentration found at time 0 for each subject.

CD4^+^ and CD8^+^ T cells were divided into naïve (Tn, CD197^+^CD45RA^+^), T effector memory (Tem, CD197^−^CD45RA^−^), T central memory (Tcm, CD197^+^CD45RA^−^), and T effector memory expressing CD45RA (Temra, CD197^−^CD45RA^+^) (Figure S3 and Table S5 in Supplementary Material). Antibodies against CD38 and HLA-DR antigens were also included in this panel to assess activation state. It was found that the administration of *L. johnsonii* N6.2 decreased the number of CD4^+^ cells after 8 weeks of treatment (*p* < 0.05), while after 12 weeks, the numbers of CD4^+^ cells were similar between the two treatment groups (*p* > 0.1) (Figure 5A; Table S5 in Supplementary Material). No changes were observed in the CD4^+^CD38^+^HLA-DR^+^ subset (Figure 5C). However, the most notable changes were obtained in the activated (CD38^+^HLA-DR^+^) CD8 T cells after 8 weeks of treatment (*p* < 0.05) (Figure 5B), as well as in the activated Temra subset which increased significantly in subjects treated with probiotic compared to placebo (*p* < 0.05) (Figure 5C). The probiotic treatment decreased the relative amount of naïve CD8^+^ T cells (*p* < 0.05) while increasing the frequency of CD8^+^ Tem (*p* < 0.05). The concentrations of both cell types were similar after the washout period (Figure 5B; Table S5 in Supplementary Material). CD4^+^ T cells showed strong trends toward decreased CD185 (CXCR5) and CD279 (PD-1) expression levels on naïve and Tem subsets as a result of the *L. johnsonii* N6.2 supplementation; however, these changes only reached statistical significance after the washout period (for CD279, *p* = 0.05 and *p* = 0.07; for CD185, *p* < 0.01 and *p* = 0.05) (Figures 6A,B). Furthermore, it was found that the administration of *L. johnsonii* N6.2 for 8 weeks significantly decreased the expression of CD279 on CD8^+^ Tem and CD8^+^ Tcm (*p* < 0.05 and *p* = 0.05, respectively) while a trend toward increased the expression of CD279 on CD8^+^ Temra cells was also observed (*p* < 0.1). These changes in CD8^+^ Tem and Tcm cells were sustained after the washout period (*p* < 0.05 and *p* < 0.05, respectively) (Figure 6C). A significant decrease of CD185 expression on CD8^+^ naïve and Tem cells was also observed at 8 weeks (*p* < 0.06 and *p* < 0.05, respectively) and sustained even after the washout period in both cell populations (*p* < 0.05 and *p* = 0.05, respectively) (Figure 6D).

#### Differentiated Teff Subsets

**Figure 7:**
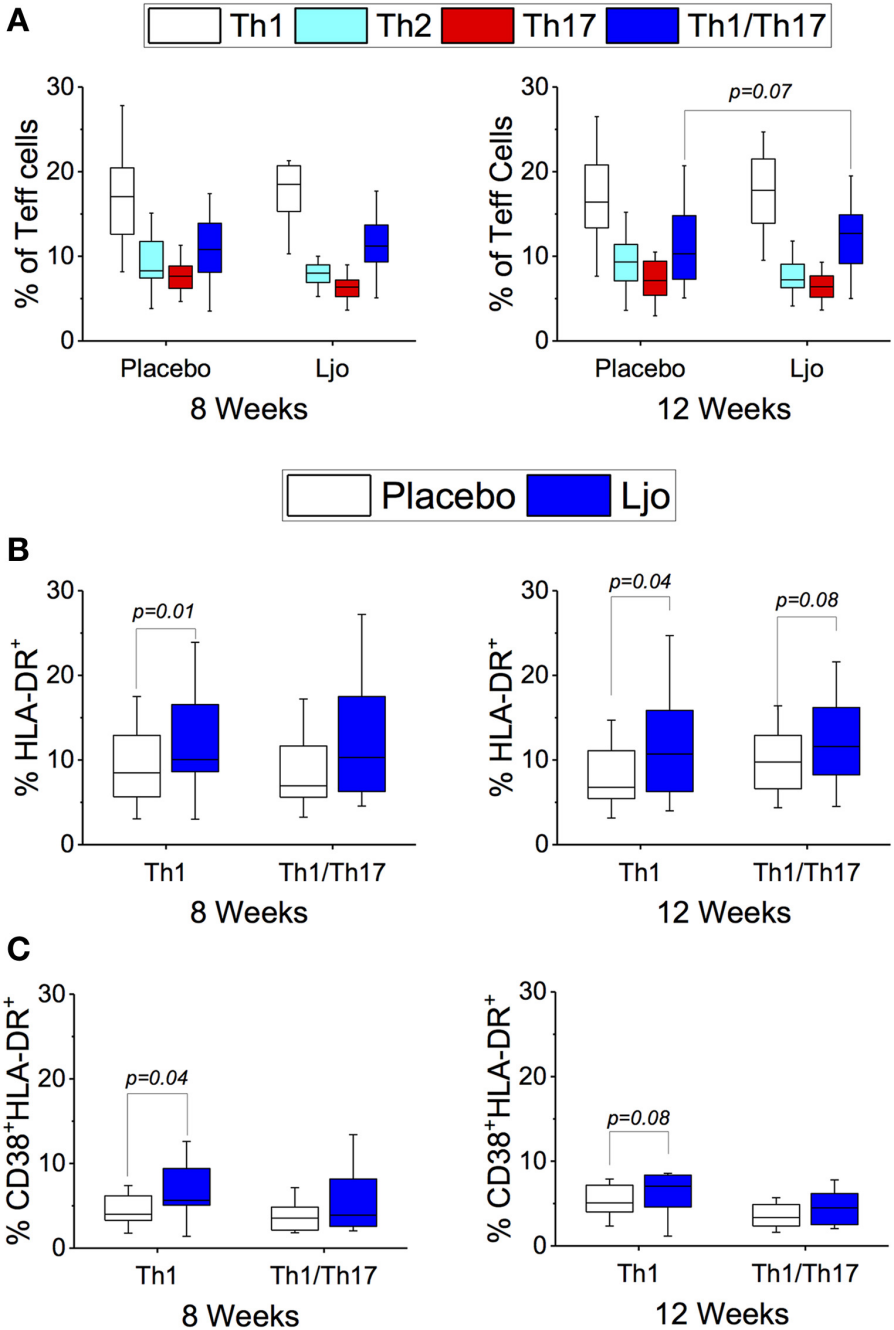
T effector cells subset (CD3^+^CD4^+^CD45RO^+^). **(A)** Th1 (CD183^+^CD196^−^), Th2 (CD183^−^CD196^−^), Th17 (CD183^−^CD196^+^), and Th1/Th17 (CD183^+^CD196^+^) were labeled with specific antibodies and quantified in the placebo (white bars) and *L. johnsonii* N6.2 (Ljo, blue bars) group at 8 and 12 weeks of treatment. **(B)** HLA-DR^+^ and **(C)** HLA-DR^+^CD38^+^ are shown for the Th1 and Th1/Th17 effector T cells. The concentration of cells shown has been normalized to the concentration found at time 0 for each subject.

CD4^+^CD45RO^+^ T cells were separated into Th1 (CD183^+^CD196^−^), Th2 (CD183^−^CD196^−^), Th17 (CD183^−^CD196^+^), and Th1/Th17 (CD183^+^CD196^+^). CD38 and HLA-DR were included to indicate activation (Figure S4 in Supplementary Material). While significant differences were not observed among the total numbers for each of the Teff subsets (Figure 7A), it was found that the number of activated Th1 (HLA-DR^+^ and HLA-DR^+^CD38^+^) were significantly increased (*p* < 0.05) in the *L. johnsonii* N6.2 treatment group (Figures 7B,C). Interestingly, the numbers remained stable after 4 weeks of washout (*p* < 0.05 and *p* < 0.09, respectively). No significant differences were observed in the activation state of the Th2, Th17, or Th1/Th17 subsets during the treatment period; however, a trend toward increased Th17 (HLA-DR^+^) and Th1/Th17 (HLA-DR^+^) cells was observed after the washout period (*p* < 0.1) (Figures 7A,B).

#### Tfh Subsets

CD4^+^CD45RA^−^CD185^+^ cells were separated into precursor (CD279^+^CD197^−^) and memory (CD279^+^CD183^−^) Tfh cells (Figure S4 in Supplementary Material). Precursor Tfh subset was significantly (*p* < 0.05) decreased in the *L. johnsonii* N6.2 group after the washout period (12 weeks). Memory Tfh was also decreased in the *L. johnsonii* N6.2 group but not statistically significant compared to placebo (Table S5 in Supplementary Material).

#### Treg Subsets

CD4^+^CD127^−/lo^CD25^+^ Tregs were separated into naïve (CD45RO^−^) and memory (CD45RO^+^). HLA-DR and CD194 expression was evaluated as well (Figure S4 in Supplementary Material). No differences were observed among the groups after 8 weeks of treatment; however, memory Tregs showed a strong trend toward increased activation (HLA-DR^+^CD194^+^) in the *L. johnsonii* N6.2 treatment group after the washout period (*p* = 0.07) (Table S5 in Supplementary Material).

### *L. johnsonii* N6.2 Increased Circulating Levels of IgA

**Figure 8:**
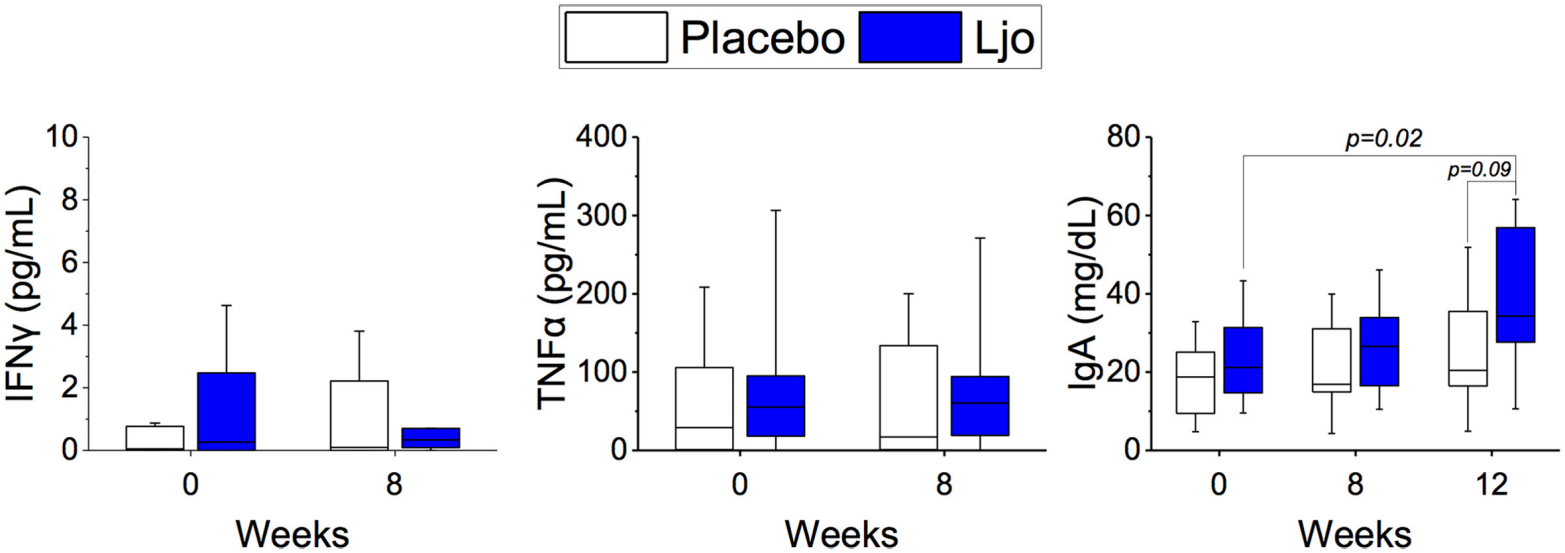
Determination of soluble markers. The concentrations of IFNγ, IgA, and TNFα were quantified in the placebo (white bars) and *L. johnsonii* N6.2 (Ljo, blue bars) group at time 0 and after 8 weeks of treatment or after the washout (12 weeks).

Based on the results obtained by immunophenotyping, we determined the levels of the following serum-soluble cytokines and immune markers: IL-6, TNF-α, IFN-γ, IFN-α, IL-2, soluble CD25 (IL-2Rα), and IgA by ELISA. IL-2 and IFN-α were below the detection limit and were not further analyzed. No statistical differences were obtained between the treatment groups or the time points for IL-6, TNF-α, IFN-γ, TNFα, and soluble CD25 (*p* > 0.1). A significant (*p* < 0.05) increase in the concentration of IgA was observed during the washout period in the *L. johnsonii* treatment group, while no differences observed in the placebo group (Figure 8).

### *L. johnsonii* N6.2 Induces Minor Changes in the Microbiota of Healthy Subjects

Based on the observations that many significant changes in the immune cell populations and IgA levels were observed 4 weeks after finalizing the *L. johnsonii* N6.2 treatment (washout period), we investigated whether *L. johnsonii* N6.2 induced changes in the microbiota.

The microbiome was analyzed at time 0 and after 8 or 12 weeks of administration of the placebo or *L. johnsonii* N6.2. DNA was extracted from all stool samples, and the microbial communities were characterized by sequencing the V4 region of the 16S rDNA with Illumina MiSeq. An average of 54,743 ± 15,800 sequencing reads per sample were obtained. Approximately 380,933 OTUs were detected, representing 173 families. The 10 most abundant families detected were *Bacteroidaceae, Lachnospiraceae, Ruminococcaceae, Prevotellaceae, Paraprevotellaceae*, an unclassified Clostridiales family, *Bifidobacteriaceae, Desulfovibrionaceae, Porphyromonadaceae*, and *Veillonellaceae* (Figure S5 in Supplementary Material), which was consistent throughout the time course of the study, although variation between individuals was observed. Bacterial communities clustered only by individual (ANOSIM *R* = 0.921, *p* < 0.01), and the community structure did not differ significantly by treatment (ANOSIM *R* = 0.011, *p* = 0.05) or over time (ANOSIM *R* = −0.017, *p* > 0.99). In addition, community structure was not correlated with the combined effects of treatment and time (PERMANOVA *R*^2^ = 0.002, *p* = 1.00) (Figure S6 in Supplementary Material). The statistical analysis showed that the relative abundance of genera or families was not significantly different between treatment groups or time points.

Due to the high variability observed in the microbiome among the subjects, we tested if the microbiome of each subject could be used to determine changes in each individual over time. Using this normalization approach, it was found that of the 173 families in the dataset, only 34 changed in relative abundance between weeks 0 and 8. The change per family was compared between the two treatment groups by Welch’s two-sample *t*-test. Although no significant differences at *p* < 0.05 were obtained, two families, *Prevotellaceae* and *Ruminococcaceae* showed trends with *p* < 0.1 (Table S6 in Supplementary Material) while families *Lactobacillaceae, Erysipelotrichaceae*, and *Odoribacteraceae* showed values of *p* = 0.17.

After the washout period, the observed microbial changes induced by *L. johnsonii* N6.2 supplementation (i.e., increase in *Ruminococcaceae, Lactobacillaceae*, and *Erysipelotrichaceae*; or decrease in *Prevotellaceae* and *Odoribacteraceae*) were reverted such that the families returned to their initial abundancies. Interestingly, one family, *Christensenellaceae* significantly increased in concentration in the *L. johnsonii* N6.2 treated group after 12 weeks (after the washout period) (*p* < 0.05), while the *Clostridiaceae* and *Bacteroidaceae* families showed trends to increase or decrease, respectively (*p* = 0.06 and *p* = 0.09, respectively). However, when this normalization method was tested at the genus level, no statistical differences were observed.

## Discussion

While the etiology of T1D is known to involve an autoimmune component, the contribution of environment to disease development remains poorly understood. However, the notion of modulating gut homeostasis with supplementation of tolerogenic “normal” commensal microbes offers a presumably safe method of intervention in the disease prevention setting. To date, a relatively limited number of studies have been performed directed at the prevention of TID, with prior trials primarily focused on nutrition-related interventions. For example, compared to standard infant formula with 20% hydrolyzed casein, the administration of hydrolyzed casein showed no significant effects on progression to autoimmunity (defined as positivity for at least two diabetes-associated autoantibodies) after 7 years of follow-up in infants at risk for T1D (39). Similarly, docosahexaenoic acid provided to at-risk infants in the first 5 months of life had no effect on inflammatory cytokine production (40).

In a recent publication from The Environmental Determinants of Diabetes in the Young (TEDDY) study group, there was a reported association between decreased risk of islet autoimmunity and early supplementation of probiotics (between the age of 0 and 27 days) when compared to no supplementation (41). Probiotics are “live microorganisms which, when administered in adequate amounts, confer a health benefit on the host” (42). A number of *Lactobacillus* and *Bifidobacterium* species are Generally Regarded as Safe microorganisms and are widely used in dietary supplements as probiotics worldwide. The mechanism of activity of probiotics is diverse and strain specific [reviewed in Ref. (43, 44)]. While the effect of different *Lactobacillus* strains on the immunological response was evaluated in several human trials (45–48), comprehensive analyses or immunophenotyping has not been performed.

Gastrointestinal microbe-based strategies for the prevention of T1D onset in humans have not yet been explored, although it has been hypothesized that the presence of certain *Lactobacillus* spp. strains may be involved in the pathogenesis of TID. *L. johnsonii* N6.2 is prevalent in BB-DR rats (16). This strain has been shown to have decreased autoimmunity onset compared to their BB-DP counterparts (14). Translating this work toward the prevention of T1D in humans first required a pilot study in healthy individuals. We performed a human trial to evaluate the safety, tolerability, and general response to consumption of this microorganism in healthy individuals. The primary outcome was the determination of safety and tolerability of oral *L. johnsonii* N6.2. Assessment of probiotic safety implicates several parameters such as immunological, microbiological, and metabolic shifts associated with the microbes’ nature, method of administration, doses, and duration of consumption (49, 50). It was found that *L. johnsonii* N6.2 preparation was well tolerated with no risks for healthy subjects. The hemogram and CMP data showed no significant differences between the probiotic and placebo groups throughout the treatment and after washout periods. No adverse events related to *L. johnsonii* N6.2 preparation were observed. Because probiotics are non-pathogenic, few of them may be related to health risks; for *Lactobacillus*, infection (lactobacillemia) is estimated to occur only once per 100 million people and has therefore been considered “unequivocally negligible” (49). *L. johnsonii* N6.2 survived intestinal transit, although no significant differences in the total numbers of LAB were observed among treatments. Results showed that *L. johnsonii* N6.2 has the ability to survive and may colonize the intestinal tract without affecting the residing microbiota in healthy subjects. Remarkably, significant changes in the kynurenine pathway metabolites as well as immune responses were observed in serum and peripheral blood, while significant changes in certain GSRS syndromes were observed in the probiotic treatment group.

Although developed for patient populations, the GSRS has been used to evaluate gastrointestinal symptoms in healthy adults (51–53). This study provides supporting data that the tool is sufficiently sensitive to detect differences in healthy individuals. A lower rating of the abdominal pain scale of the GSRS was demonstrated for *L. johnsonii* N6.2 versus placebo. The individual symptom data suggest that *L. johnsonii* N6.2 may demonstrate a beneficial effect by reducing stomach ache or pain in the healthy adults studied, but the strong trend for significance suggests that the groups may have differed at baseline and thus, this may also be a carryover effect. However, the data suggest that the difference between the groups may have increased with *L. johnsonii* N6.2. Although this potential mitigating effect on abdominal pain has not been reported previously for *L. johnsonii*, other *Lactobacillus* spp. have been evaluated for efficacy in improving abdominal pain. For *L. rhamnosus* GG (LGG), the reported effects in human trials were inconsistent. Francavilla et al. (54) reported that LGG was effective at 3 × 10^9^ to 1 × 10^10^ CFU/day on reducing abdominal pain severity/intensity or frequency in children with irritable bowel syndrome (IBS) and functional abdominal pain (FAP). In addition, Gawrońska et al. (55) reported that LGG may moderately increase treatment success without effect on pain severity or may result in no differences at all compared to placebo according to Bauserman and Michail (56). Conversely, *Lactobacillus reuteri* showed no improvement over placebo in FAP in children (57). In adults with IBS, provision of *Lactobacillus plantarum* 299v at a dose of 1 × 10^10^ CFU/day showed significantly lower abdominal pain severity and frequency (58). Similarly, *Lactobacillus casei rhamnosus* at 6 × 10^8^ CFU/day demonstrated a clinically significant improvement in abdominal pain in a subgroup of IBS patients with a predominance of diarrhea, although small sample size precluded statistical analysis (59). By contrast, *L. reuteri* was ineffective in lessening abdominal pain in IBS patients (60). As mitigation of abdominal pain may be strain specific, the results of this study suggest that further research is needed to explore the potential efficacy of *L. johnsonii* N6.2 in individuals with abdominal pain such as those with IBS.

Of interest is the effectiveness of *L. johnsonii* N6.2 on lessening the daily symptom reporting of headache and cramping. Very little research has explored the effect of probiotic supplementation on headache. In a dose–response trial of healthy adults, the effect of *Bifidobacterium animalis* subspecies *lactis* (BB-12) and *Lactobacillus paracasei* subspecies *paracasei* (CRL431) on well-being including the symptoms of bloating, flatulence, and headache were evaluated, but no changes in the interventions were observed (61). In regards to cramping, a 4-week intervention of *Lactobacillus acidophilus* DDS-1 improved abdominal cramping with a lactose challenge in adults with lactose intolerance (62). Further research is needed to determine if *L. johnsonii* N6.2 is effective in mitigating headache and cramping in clinical populations.

In BB-DR rats, the mechanism of *L. johnsonii*–host interactions related to prevention of TID may involve downregulation of the production of kynurenine and increasing tryptophan flux toward the synthesis of serotonin (25). To evaluate whether or not these fluctuations in tryptophan metabolites observed in rats could be used as a marker of *L. johnsonii* activity in humans, the levels of tryptophan metabolites were determined in serum throughout the study. A strong trend toward decreased serum levels of kynurenine along with increased amounts of tryptophan was observed in a subgroup of participants who consumed *L. johnsonii* N6.2 and exhibited an increase of LAB CFU/g stool over the treatment period. These fluctuations in the metabolites may be related to IDO activity, which has been associated with immune regulation and modulation of chronic inflammation, as well as allergic and autoimmune disorders. Many studies have focused on the inhibition of IDO to regulate effector metabolites as kynurenine derivate, serotonin, and tryptophan [for a review, see Ref. (21)]. Our group originally described the use of *L. johnsonii* N6.2 to modulate IDO activity *in vitro* and *in vivo* (25). In a recent study, the administration of commercial probiotics (Vivomixx in Europe or Visbiome in USA) to HIV^+^ patients decreased the IDO mRNA expression levels in gut-associated lymphoid tissue after 6 months of administration. In these patients, IDO was overexpressed in the gut mucosa, and it was proposed that the downregulation of IDO would be necessary to decrease its harmful effects on the mucosal barrier (63). In this study, we determined the concentrations of several intermediates in the tryptophan degradation pathway. Although statistical significance was not achieved for any metabolite during the treatment period, a significant increase in the tryptophan concentrations and a decrease in the tryptophan/kynurenine ratio were observed after the washout period (12 weeks) in the *L. johnsonii* N6.2 group. An interesting finding of this study was that the symptom of anxiety was significantly lower in the *L. johnsonii* N6.2 group during the washout period. This symptom has been associated with decreased levels of serotonin (64, 65), and since we observed differences in the symptom score in the *L. johnsonii* N6.2 group, we speculate that the modulation of IDO activity by the probiotic may have channeled the tryptophan concentrations toward the production of serotonin. However, the serotonin levels were highly variable, and no statistical differences were observed.

Modification in the activity of IDO activity in antigen-presenting cells (APCs), such as DCs and macrophages, or NK cells has been reported to have a broad impact on the immune system directly affecting T cells (22, 23, 66). Previous reports have shown that several species of LAB may exert direct effects on APCs (DC cells and monocytes), macrophages, and to a lesser extent on B cells (67–71). To assess the global impact of *L. johnsonii* N6.2’s modifications of IDO, we performed immunophenotyping and quantified the relative concentrations of B cells, Monocytes, NK and T cells (including Teff, Treg, and Tfh). The administration of *L. johnsonii* N6.2 resulted in a progressive increase in the frequencies of monocytes and NK cells (specifically the activated NK CD16^+^CD56^hi^ subset), reaching statistical significance after the washout period. However, B cells or DCs did not show differences between the groups during the treatment period or after washout. NK cell activation may result from cell-to-cell contact as result of NK/DC cross talk (72) or it may result from to the direct interaction of *L. johnsonii* N6.2 associated molecules, such as lipids or DNA, with NK cells. As we have previously observed *in vitro* that *L. johnsonii* N6.2 can stimulate the innate immune response through TLR9 signaling (73), we hypothesize that TLR9 activation is a likely mechanism.

Mailliard et al. (74) reported that the interaction of NK cells with DCs contributed to the maturation of Th1 cells and IFN-γ^+^CD8^+^ T cells. In this study, we observed a significant increase in the activated HLA-DR^+^CD38^+^ Th1 population after the 8 weeks of treatment and after the washout (12 weeks). These results are in agreement with previous reports seeking allergy treatments where it was observed that the administration of *L. paracasei* induced a Th1 type response in mice (75).

Notably, we observed significant changes in most CD8^+^ T cells subsets: Tn, Tcm, Tem, and Temra. Among them, activated CD38^+^HLA-DR^+^ CD8^+^ T cells increased significantly at 8 weeks and after washout (12 weeks). These results are in agreement with the activation of Th1 responses mediated by NK cells. The decrease in CD8^+^ Tn and increase in CD8^+^ Tem cells observed in this study has been reported previously after antigenic stimulation (Type 1 response) where Tn cells differentiated into two main subsets as Tem and Tcm cells (76). However, in the previous study, after antigen stimulation ceased, most of the CD8^+^ Teff cells decreased by apoptosis, while a small percentage remained as mature memory CD8^+^ T cells (77). Here, we observed that the populations remained either decreased or increased after the washout period. This observation coincided with a significantly decrease expression of the inhibitory coreceptor, CD279^+^ (PD-1), and of the follicular homing chemokine receptor, CD185^+^ (CXCR5), on CD8^+^ Tem in the *L johnsonii* N6.2 group after 8 weeks of treatment. These results indicate that *L. johnsonii* N6.2 may reduce or delay apoptosis of memory CD8^+^ T cells.

In summary, we identified systemic biomarkers [such as the increase of circulating effector Th1 cells (CD45RO^+^CD183^+^CD196^−^) and cytotoxic CD8^+^ T cells] that can be utilized to follow the effects of *L. johnsonii* N6.2 consumption in healthy subjects. The results of this pilot study provide a solid foundation for an investigation into prevention of T1D onset by *L. johnsonii* N6.2 in an at-risk human population.

## Ethics Statement

This study was carried out in accordance with the recommendations of the Institutional Review Board (# 201400370) at the University of Florida with written informed consent from all subjects. All subjects gave written informed consent in accordance with the Declaration of Helsinki. The protocol was approved by the Institutional Review Board at University of Florida (this trial was registered at http://clinicaltrials.gov as NCT02349360).

## Author Contributions

GM, AF, DC, and NH performed research. GM, AF, SG, DC, NH, and JM analyzed data. WD, DP, TG, TB, MA, MH, CG, and CW contributed to discussion and reviewed the manuscript. WD and GL conceived the study.

## Conflict of Interest Statement

Authors, GL and MA, hold U.S. Patent No. 9,474,773. The other authors declare that the research was conducted in the absence of any commercial or financial relationships that could be construed as a potential conflict of interest.

## Supplementary Material

Figure S1 Flow cytometry gating strategy for evaluation of the B cells subset in healthy subjects.

Figure S2 Flow cytometry gating strategy for evaluation of the NK, monocytes and dendritic cells subsets in healthy subjects.

Figure S3 Flow cytometry gating strategy for evaluation of naïve and memory T cells subsets in healthy subjects.

Figure S4 Flow cytometry gating strategy for evaluation of differentiated effector T (Teff), T follicular helper, and regulatory T (Treg) cells subsets in healthy subjects.

Figure S5 Relative abundance of bacterial families in gut microbiota. The 10 most abundant families in stool samples from subjects given a placebo **(A)** or *Lactobacillus johnsonii* N6.2 **(B)** treatment. The *Lactobacillaceae* family was also included for comparison.

Figure S6 Non-metric multidimensional scaling plot of microbial community similarity based on Bray–Curtis beta diversity of Illumina MiSeq 16S rRNA gene libraries. Points shown belong to the sampling time points: T0, T1 = 2 weeks, T2 = 4 weeks, T3 = 8 weeks (end of treatment), and T4 = 12 weeks. Polygons connect all the samples for one subject, and the bounding lines are colored according to the treatment group.
