# Supplementary figures and images for: Lactobacillus johnsonii N6.2 Modulates the Host Immune Responses: A Double-Blind, Randomized Trial in Healthy Adults
Source: Front Immunol. 2017 Jun 12;8:655. doi: 10.3389/fimmu.2017.00655 (PMC5466969; doi:10.3389/fimmu.2017.00655)

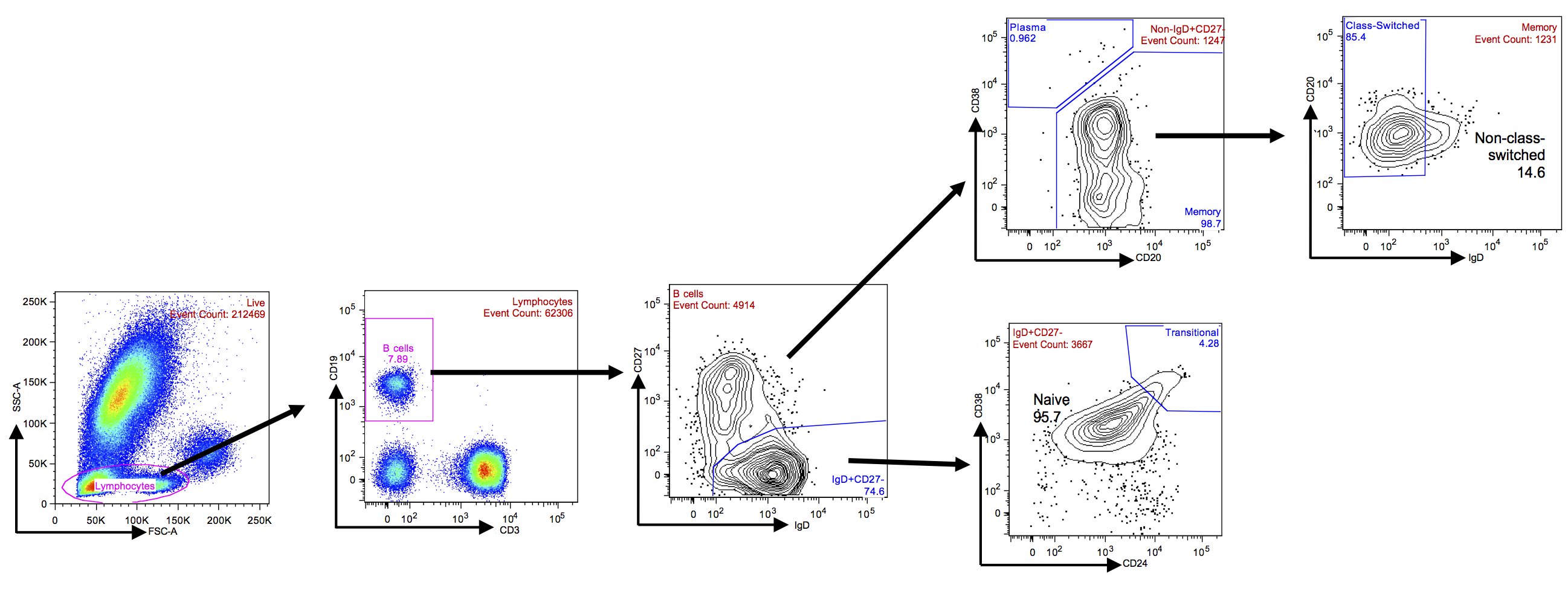

Supplement: Figure S1 — Flow cytometry gating strategy for evaluation of the B cells subset in healthy subjects. [file Image_1.TIFF]

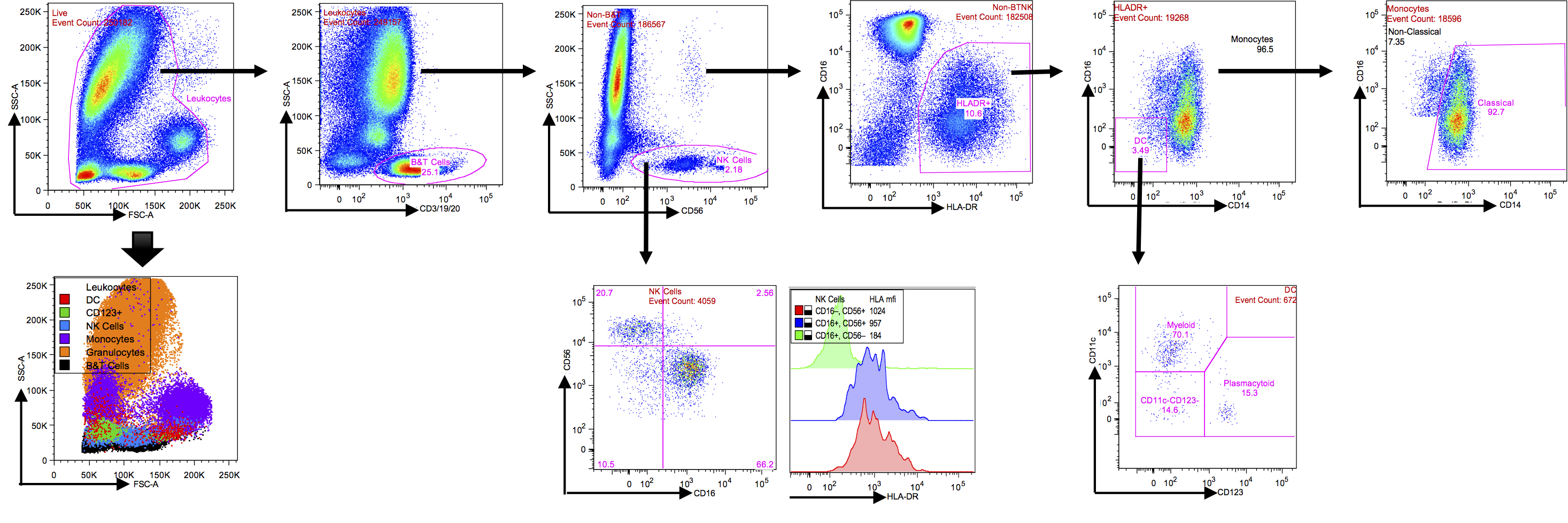

Supplement: Figure S2 — Flow cytometry gating strategy for evaluation of the NK, monocytes and dendritic cells subsets in healthy subjects. [file Image_2.TIFF]

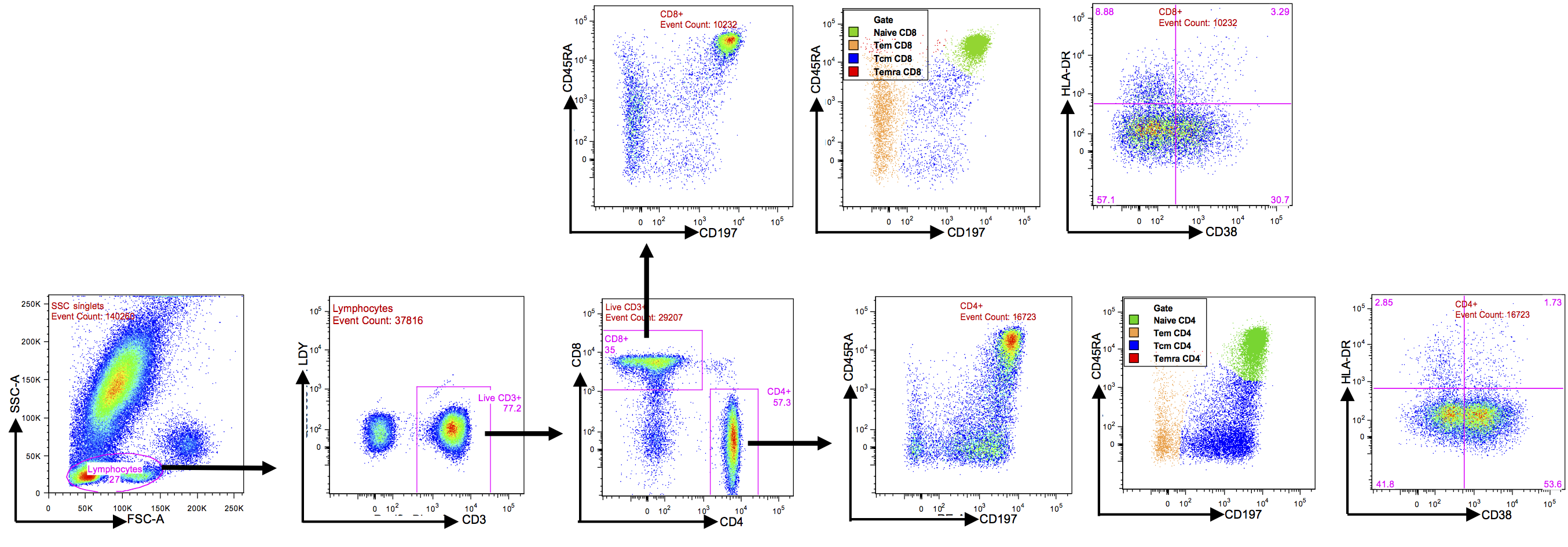

Supplement: Figure S3 — Flow cytometry gating strategy for evaluation of naïve and memory T cells subsets in healthy subjects. [file Image_3.TIFF]

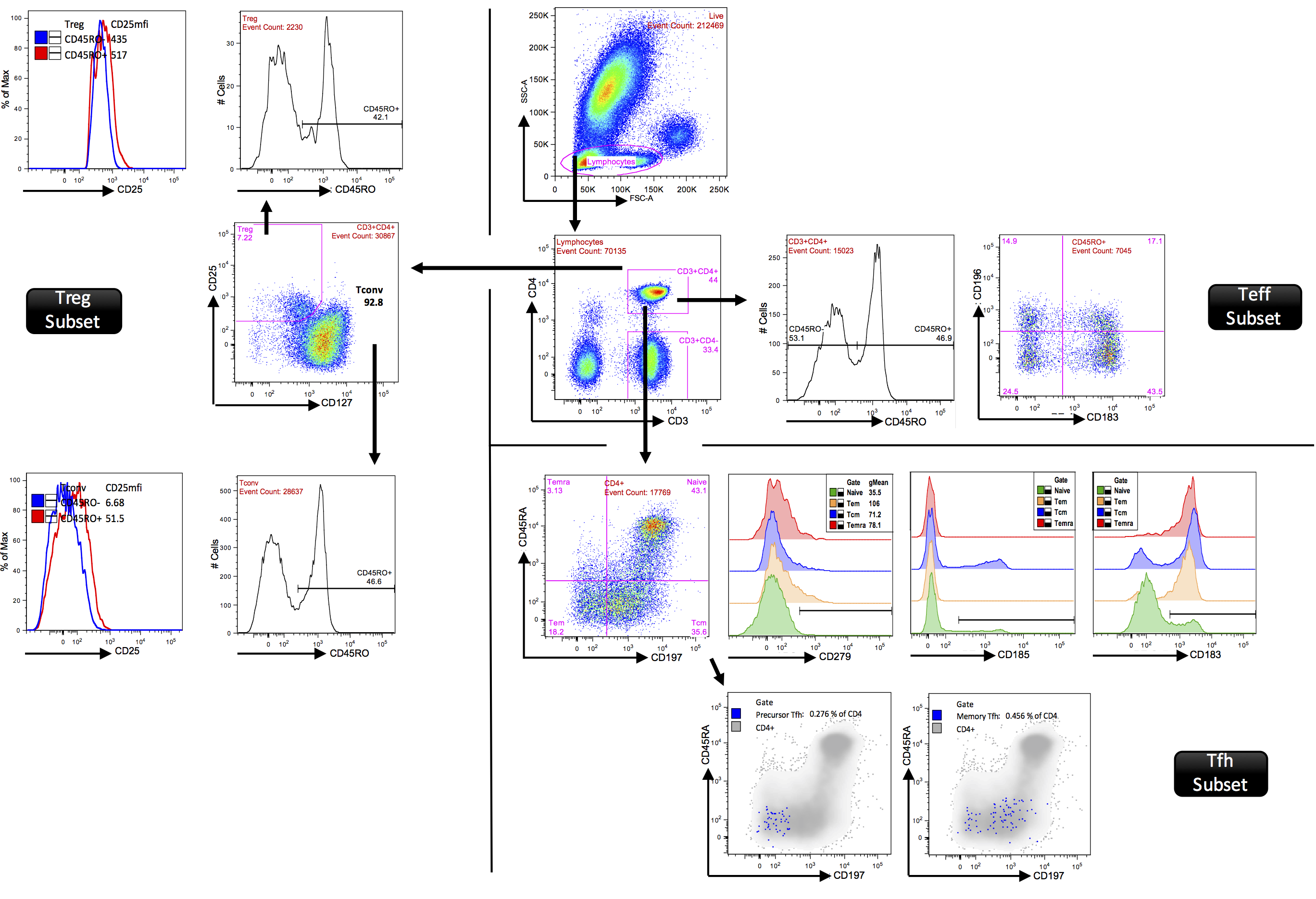

Supplement: Figure S4 — Flow cytometry gating strategy for evaluation of differentiated effector T (Teff), T follicular helper, and regulatory T (Treg) cells subsets in healthy subjects. [file Image_4.TIFF]

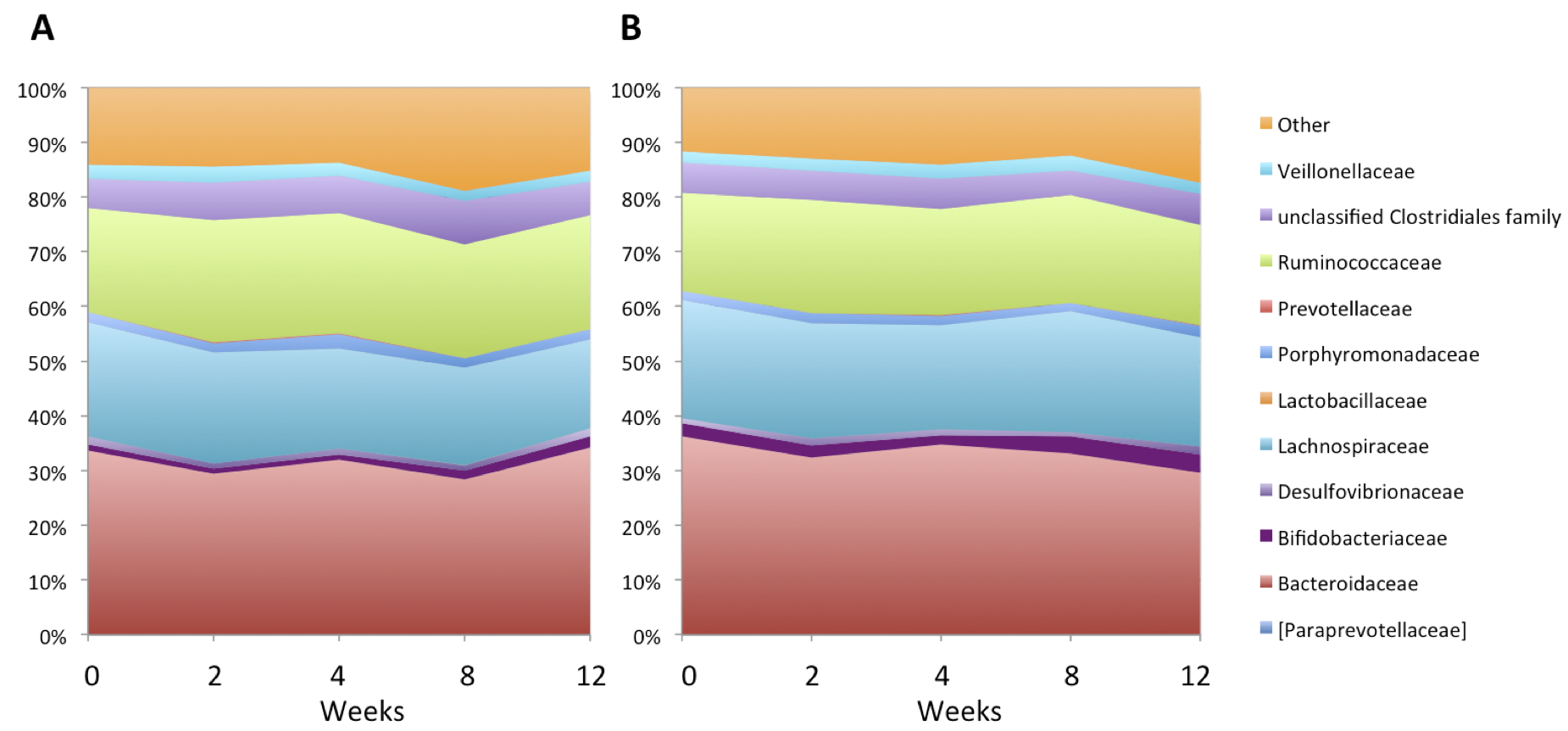

Supplement: Figure S5 — Relative abundance of bacterial families in gut microbiota. The 10 most abundant families in stool samples from subjects given a placebo (A) or Lactobacillus johnsonii N6.2 (B) treatment. The Lactobacillaceae family was also included for comparison. [file Image_5.TIFF]

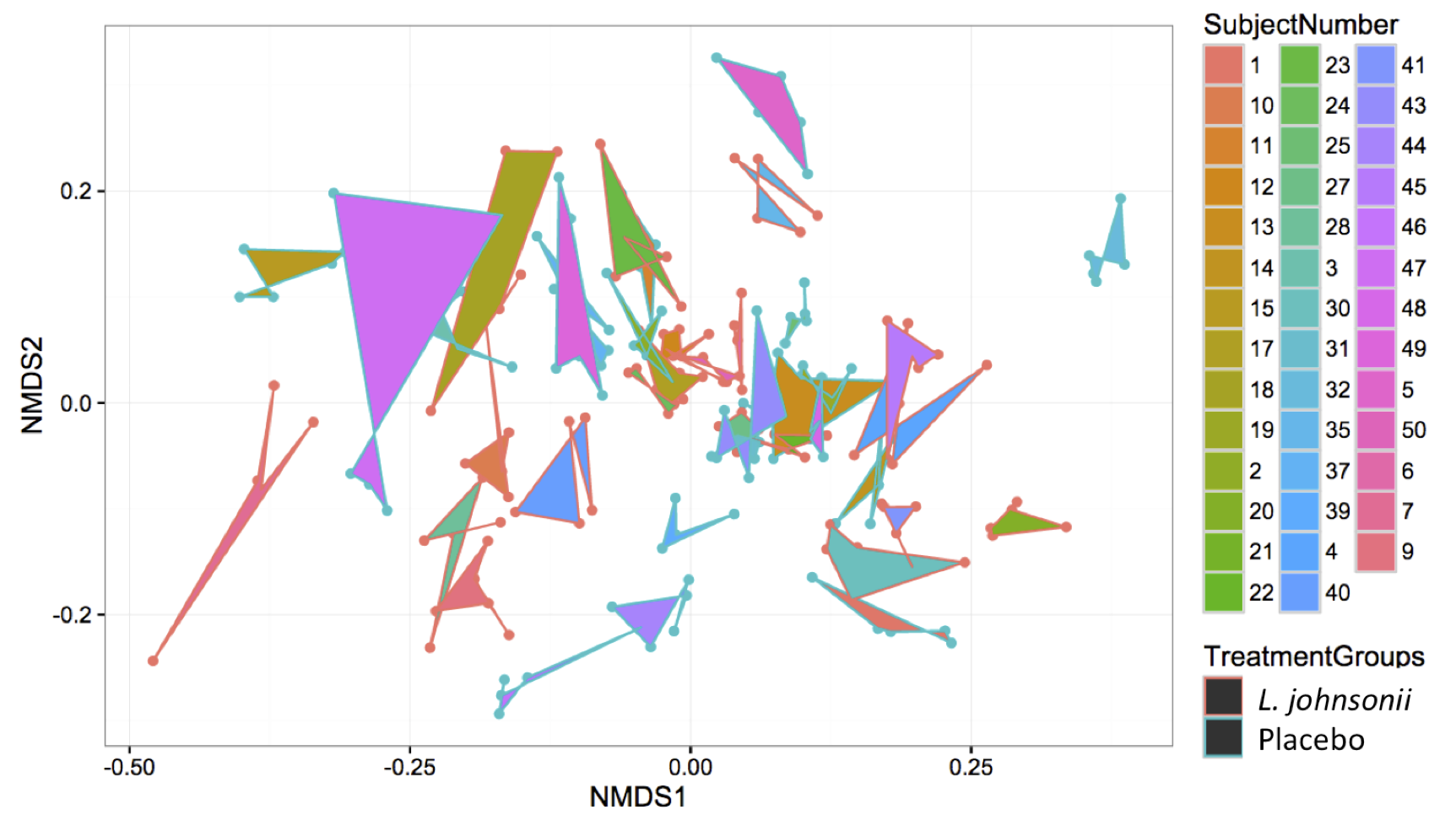

Supplement: Figure S6 — Non-metric multidimensional scaling plot of microbial community similarity based on Bray–Curtis beta diversity of Illumina MiSeq 16S rRNA gene libraries. Points shown belong to the sampling time points: T0, T1 = 2 weeks, T2 = 4 weeks, T3 = 8 weeks (end of treatment), and T4 = 12 weeks. Polygons connect all the samples for one subject, and the bounding lines are colored according to the treatment group. [file Image_6.TIFF]
