# Supplementary material for: Lactobacillus johnsonii N6.2 Modulates the Host Immune Responses: A Double-Blind, Randomized Trial in Healthy Adults
Source: Front Immunol. 2017 Jun 12;8:655. doi: 10.3389/fimmu.2017.00655 (PMC5466969; doi:10.3389/fimmu.2017.00655)
Supplement: Supplementary file 7 [file Table_1.DOCX]

**Supplementary Table 1**. **Summary of the results obtained for relevant indicators of kidney and liver function.**

| **Measure** | **Normal Values** | **Placebo (n=20)** | | | | |
| --- | --- | --- | --- | --- | --- | --- |
|  |  | **Weeks** | | | | |
|  |  | **0** | **2** | **4** | **8** | **12** |
| **Glucose (mg/dL)** | 70-100 | 80.0±7.9 | 84.5±1.5 | 84.6±1.7 | 83.4±1.7 | 83.2±1.6 |
| **KIDNEY FUNCTION** | | | | | | |
| **Urea (mg/dL)** | 7-20 | 12.6±4.9 | 13.8±0.5 | 14.4±0.7 | 13.7±0.6 | 12.7±0.6 |
| **Creatinine (mg/dL)** | 0.8-1.4 | 0.85±0.28 | 0.81±0.01 | 0.81±0.02 | 0.77±0.02 | 0.76±0.01 |
| **LIVER FUNCTION** | | | | | | |
| **Aspartate Aminotransferase (IU/L)** | 10-34 | 25.8±7.2 | 23.2±1.0 | 26.8±1.5 | 25.1±1.4 | 24.7±1.4 |
| **Alanine Aminotransferase (IU/L)** | 8-37 | 19.9±8.3 | 18.5±1.2 | 22.3±1.8 | 21.1±2.1 | 18.9±2.0 |
| **Alkaline Phosphatase (IU/L)** | 44-147 | 63.4±14.6 | 66.9±1.6 | 64.5±1.9 | 71.1±2.1 | 71.7±2.0 |
| **Total Bilirubin (mg/dL)** | 0.2-1.9 | 0.53±0.18 | 0.60±0.05 | 0.63±0.04 | 0.54±0.05 | 0.58±0.06 |

| **Measure** | **Normal Values** | ***L. johnsonii* N6.2 (n=21)** | | | | |
| --- | --- | --- | --- | --- | --- | --- |
|  |  | **Weeks** | | | | |
|  |  | **0** | **2** | **4** | **8** | **12** |
| **Glucose (mg/dL)** | 70-100 | 80.9±8.4 | 84.3±1.5 | 84.6±1.7 | 84.9±1.7 | 85.3±1.6 |
| **KIDNEY FUNCTION** | | | | | | |
| **Urea (mg/dL)** | 7-20 | 12.8±3.4 | 13.9±0.5 | 15.1±0.8 | 14.3±0.6 | 13.8±0.6 |
| **Creatinine (mg/dL)** | 0.8-1.4 | 0.79±0.16 | 0.83±0.01 | 0.81±0.02 | 0.78±0.02 | 0.76±0.01 |
| **LIVER FUNCTION** | | | | | | |
| **Aspartate Aminotransferase (IU/L)** | 10-34 | 25.2±6.9 | 26.0±1.0 | 24.1±1.4 | 27.1±1.4 | 25.7±1.4 |
| **Alanine Aminotransferase (IU/L)** | 8-37 | 16.2±5.6 | 19.7±1.3 | 18.9±1.7 | 20.8±2.1 | 21.1±2.0 |
| **Alkaline Phosphatase (IU/L)** | 44-147 | 69.1±17.4 | 65.2±1.6 | 69.0±1.9 | 69.3±2.1 | 68.6±2.0 |
| **Total Bilirubin (mg/dL)** | 0.2-1.9 | 0.52±0.12 | 0.65±0.05 | 0.59±0.04 | 0.70±0.05 | 0.64±0.06 |
