# Supplementary material for: Lactobacillus johnsonii N6.2 Modulates the Host Immune Responses: A Double-Blind, Randomized Trial in Healthy Adults
Source: Front Immunol. 2017 Jun 12;8:655. doi: 10.3389/fimmu.2017.00655 (PMC5466969; doi:10.3389/fimmu.2017.00655)
Supplement: Supplementary file 8 [file Table_2.DOCX]

**Supplementary Table 2. Select individual symptoms of the daily and weekly questionnaires.**

| **Period** | **Stomachache or pain^w^** | | **Bloating^d^** | | **Cramping^d^** | | **Abdominal Noises^d^** | | **Headache^d^** | | **Anxiety^d^** | |
| --- | --- | --- | --- | --- | --- | --- | --- | --- | --- | --- | --- | --- |
|  | **Placebo** | **Ljo** | **Placebo** | **Ljo** | **Placebo** | **Ljo** | **Placebo** | **Ljo** | **Placebo** | **Ljo** | **Placebo** | **Ljo** |
| **Baseline** | 1.7±0.1 | 1.0±0.1 | 0.4±0.05 | 0.1±0.05 | 0.3±0.05 | 0.01±0.05 | 0.3±0.05 | 0.06±0.05 | 0.4±0.04 | 0.1±0.04 | 0.5±0.1 | 0.3±0.1 |
| **Week 1** | 1.9±0.2 | 1.2±0.2* | 0.4±0.1 | 0.2±0.1 | 0.4±0.1 | 0.1±0.1** | 0.3±0.1 | 0.1±0.1 | 0.3±0.1 | 0.4±0.1 | 0.3±0.2 | 0.6±0.2 |
| **Week 2** | 2.3±0.2 | 1.0±0.2*** | 0.3±0.1 | 0.2±0.1 | 0.3±0.1 | 0.01±0.1** | 0.3±0.1 | 0.1±0.1 | 0.7±0.1 | 0.2±0.1** | 0.5±0.2 | 0.3±0.2 |
| **Week 3** | 1.5±0.2 | 1.1±0.2* | 0.3±0.1 | 0.05±0.1** | 0.4±0.1 | 0.01±0.1** | 0.4±0.1 | 0.04±0.1* | 0.3±0.1 | 0.2±0.1 | 0.4±0.1 | 0.2±0.1 |
| **Week 4** | 1.5±0.1 | 1.0±0.1** | 0.6±0.1 | 0.1±0.1** | 0.2±0.1 | 0.01±0.1** | 0.3±0.1 | 0.1±0.1* | 0.4±0.1 | 0.04±0.1** | 0.4±0.2 | 0.4±0.2 |
| **Week 5** | 1.4±0.1 | 0.9±0.1* | 0.4±0.1 | 0.04±0.1** | 0.1±0.1 | 0.04±0.1 | 0.3±0.1 | 0.1±0.1* | 0.4±0.1 | 0.2±0.1 | 0.4±0.1 | 0.3±0.1 |
| **Week 6** | 1.4±0.1 | 1.0±0.1* | 0.2±0.1 | 0.2±0.1 | 0.2±0.05 | 0.01±0.05* | 0.2±0.1 | 0.02±0.1 | 0.5±0.1 | 0.02±0.1** | 0.5±0.2 | 0.2±0.2 |
| **Week 7** | 1.8±0.2 | 0.9±0.2*** | 0.3±0.1 | 0.1±0.1 | 0.3±0.1 | 0.01±0.1** | 0.3±0.1 | 0.01±0.1** | 0.4±0.1 | 0.01±0.1*** | 0.5±0.2 | 0.2±0.2 |
| **Week 8** | 1.8±0.2 | 1.0±0.2** | 0.3±0.2 | 0.05±0.1* | 0.2±0.1 | 0.01±0.2** | 0.3±0.1 | 0.01±0.1** | 0.2±0.05 | 0.02±0.1** | 0.5±0.2 | 0.2±0.2 |
| **Washout 1** | 1.6±0.2 | 1.0±0.2* | 0.5±0.1 | 0.3±0.2* | 0.2±0.1 | 0.01±0.1** | 0.2±0.1 | 0.01±0.1** | 0.2±0.1 | 0.03±0.1 | 0.5±0.1 | 0.1±0.1* |
| **Washout 2** | 1.5±0.2 | 1.0±0.2 | 0.6±0.1 | 0.1±0.1** | 0.3±0.1 | 0.01±0.1* | 0.3±0.1 | 0.01±0.1* | 0.4±0.1 | 0.01±0.1*** | 0.5±0.1 | 0.1±0.1* |
| **Washout 3** | 1.9±0.2 | 1.0±0.2** | 0.6±0.1 | 0.1±0.1** | 0.5±0.2 | 0.01±0.2 | 0.4±0.2 | 0.04±0.2 | 0.3±0.2 | 0.1±0.2 | 0.5±0.2 | 0.1±0.1 |
| **Washout 4** | 1.6±0.1 | 1.0±0.1* | 0.5±0.1 | 0.02±0.1** | 0.2±0.1 | 0.01±0.1* | 0.3±0.1 | 0.01±0.1* | 0.3±0.1 | 0.1±0.1 | 0.6±0.2 | 0.2±0.1* |

Ljo correspond to *Lactobacillus johnsonii* N6.2.

Data presented as Least Squares mean ± SEM. **p*<0.05; ***p*<0.01; ****p*<0.001.

^w^= from weekly questionnaire; ^d^= from daily questionnaire.
