# Supplementary material for: Lactobacillus johnsonii N6.2 Modulates the Host Immune Responses: A Double-Blind, Randomized Trial in Healthy Adults
Source: Front Immunol. 2017 Jun 12;8:655. doi: 10.3389/fimmu.2017.00655 (PMC5466969; doi:10.3389/fimmu.2017.00655)
Supplement: Supplementary file 9 [file Table_3.DOCX]

**Supplementary Table 3. Summary of the concentrations of metabolites obtained within the tryptophan pathway.**

| **Metabolite^1^** | **Placebo (n=20)** | | | | |
| --- | --- | --- | --- | --- | --- |
|  | **Weeks** | | | | |
| **(nMol)** | 0 | 2 | 4 | 8 | 12 |
| **Serotonine** | 0.024±0.022 | 0.026±0.013 | 0.026±0.009 | 0.041±0.015 | 0.049±0.014 |
| **Kynurenine** | 1.90±0.40 | 2.00±0.10 | 2.00±0.08 | 2.01±0.08 | 1.97±0.14 |
| **Tryptophan** | 61.2±8.2 | 63.3±2.4 | 65.2±3.0 | 63.9±2.5 | 60.3±5.7 |
| **Xanthurenic acid** | 0.015±0.006 | 0.018±0.002 | 0.018±0.002 | 0.017±0.002 | 0.014±0.003 |
| **Kynurenic acid** | 0.079±0.04 | 0.110±0.010 | 0.103±0.009 | 0.106±0.011 | 0.092±0.010 |
| **K:T (x1000)^2^** | 31.5±5.4 | 32.6±1.2 | 31.3±1.2 | 32.3±1.1 | 33.3±2.2 |
|  | | | | | |
| **Metabolite^1^** | ***L. johnsonii* N6.2 (n=21)** | | | | |
|  | **Weeks** | | | | |
| **(nMol)** | 0 | 2 | 4 | 8 | 12 |
| **Serotonine** | 0.022±0.016 | 0.036±0.013 | 0.021±0.009 | 0.036±0.014 | 0.022±0.015 |
| **Kynurenine** | 2.0±0.4 | 2.1±0.1 | 2.12±0.09 | 2.14±0.08 | 2.30±0.15 |
| **Tryptophan** | 61.0±10.8 | 66.0±2.5 | 68.7±3.2 | 67.1±2.5 | 70.6±6.1 |
| **Xanthurenic acid** | 0.017±0.008 | 0.019±0.002 | 0.020±0.002 | 0.020±0.002 | 0.020±0.003 |
| **Kynurenic acid** | 0.097±0.005 | 0.114±0.010 | 0.109±0.009 | 0.128±0.011 | 0.116±0.010 |
| **K:T (x1000) ^2^** | 33.3±6.6 | 32.3±1.2 | 31.9±1.2 | 32.1±1.1 | 34.1±2.4 |

^1^ Anthranilic acid was also determined however the values obtained were below the detection limit of the instrument (3 ng/mL).

^2^ Ratio kynurenine: tryptophan
