# Supplementary material for: Lactobacillus johnsonii N6.2 Modulates the Host Immune Responses: A Double-Blind, Randomized Trial in Healthy Adults
Source: Front Immunol. 2017 Jun 12;8:655. doi: 10.3389/fimmu.2017.00655 (PMC5466969; doi:10.3389/fimmu.2017.00655)
Supplement: Supplementary file 10 [file Table_4.DOCX]

**Supplementary Table 4. B cells, Monocytes, Dendritic cells and Natural Killer subset.**

|  |  |  | **8 weeks** | | **12 weeks** | |
| --- | --- | --- | --- | --- | --- | --- |
| **Lymphocytes** | **Subpopulations** |  | **Placebo** | **Ljo** | **Placebo** | **Ljo** |
| **B cells %**  **(CD3^-^CD19^+^)** |  |  | 10.7±0.4 | 10.9±0.4 | 11.8±0.4 | 11.3±0.4 |
|  | Transitional %  (CD27^-^IgD^+^CD24^hi^CD38^hi^) |  | 3.±0.3 | 3.2±0.3 | 2.8±0.3 | 3.5±0.3 |
|  | Naïve %  (CD27^-^IgD^+^CD24^lo/-^CD38^lo/-^) |  | 67.3±1.3 | 68.3±1.3 | 65.8±1.1 | 67.4±1.1 |
|  | Non-class-switched Memory % (CD20^hi^CD27^+^IgD^+^) |  | 8.7±1.0 | 7.1±1.0 | 10.7±1.0 | 8.4±1.0^Ψ^ |
|  | Class-switched Memory %  (CD20^hi^CD27^+^IgD^-^) |  | 20.3±1.1 | 21.4±1.1 | 20.2±0.8 | 20.7±0.9 |
|  | Plasmablast  (CD20^lo/-^CD38^+^) |  | 0.56±0.10 | 0.36±0.10 | 0.36±0.08 | 0.41±0.08 |
| **Granulocytes %** |  |  | 47.1±1.7 | 47.8±1.7 | 50.6±1.8 | 51.8±1.8 |
| **MNC %**  **(non-granulocytes)**  **(CD3^-^CD19^-^CD20^-^)** |  |  | 45.90±2.1 | 48.8±2.1 | 43.9±1.8 | 41.5±1.8 |
|  | DC % (of MNC)  (HLA-DR^+^CD14^-^CD16^-^) |  | 0.89±0.18 | 1.01±0.17 | 1.04±0.32 | 1.05±0.31 |
|  |  | HLA-DR mfi | 10966±598 | 11068±606 | 12369±605 | 12335±612 |
|  |  | Myeloid %  (CD11c^+^CD123^-^) | 63.2±1.8 | 64.8±1.8 | 66.0±1.7 | 65.6±1.7 |
|  |  | Plasmacytoid %  (CD123^+^) | 22.5±1.8 | 21.9±1.8 | 22.5±1.3 | 23.0±1.3 |
|  | Monocytes %  (HLA-DR^+^CD14^+^ CD56^-^) |  | 16.5±0.9 | 17.1±0.9 | 16.1±1.1 | 19.7±1.1* |
|  |  | HLA-DR mfi | 6423±305 | 5855±308 | 6015±369 | 6209±368 |
|  |  | Classical %  (CD16^-^) | 88.3±1.0 | 88.4±1.0 | 90.5±0.7 | 89.2±0.7 |
|  |  | Non-Classical % (CD16^+^) | 11.6±1.0 | 11.6±1.0 | 9.5±0.7 | 10.8±0.7 |
| **NK %**  **(of lymphocytes)**  **(CD3^-^CD19^-^CD14^-^)** |  |  | 9.2±0.7 | 10.6±0.7 | 8.4±0.7 | 10.4±0.8^Ψ^ |
|  | CD16^-^CD56^hi^ % |  | 6.3±0.7 | 5.7±0.7 | 5.7±0.6 | 5.7±0.6 |
|  | CD16^+^CD56^lo/-^ % |  | 84.8±1.0 | 85.9±1.0 | 84.9±1.0 | 84.8±1.0 |
|  | CD16^+^CD56^hi^ % |  | 2.8±0.4 | 2.2±0.4 | 3.0±0.2 | 2.5±0.2 |

B cells (transitional, memory and plasmablast), Mononuclear cells (DC, Myeloid, Plasmacytoid and monocytes) and NKs were analyzed by multicolor flow cytometry using specific antibodies for CD3, CD11c, CD14, CD16, CD19, CD20, CD24, CD27, CD38, CD56 and CD123. The populations were analyzed after 8 and 12 week of consumption of the placebo or *L. johnsonii* N6.2 (Ljo). Data presented as Least Squares mean ± SEM. ^Ψ^*p*<0.1; **p*<0.05; ***p*<0.01.
