# Supplementary material for: Lactobacillus johnsonii N6.2 Modulates the Host Immune Responses: A Double-Blind, Randomized Trial in Healthy Adults
Source: Front Immunol. 2017 Jun 12;8:655. doi: 10.3389/fimmu.2017.00655 (PMC5466969; doi:10.3389/fimmu.2017.00655)
Supplement: Supplementary file 11 [file Table_5.DOCX]

**Supplementary Table 5. T cells subset: CD4^+^ and CD8^+^, Follicular helper, Regulatory and Conventional T cells were defined.**

|  |  |  | **8 weeks** | | **12 weeks** | |
| --- | --- | --- | --- | --- | --- | --- |
| **Lymphocytes** | **Subpopulations** |  | **Placebo** | **Ljo** | **Placebo** | **Ljo** |
| **T cells %**  **(CD3^+^)** |  |  | 72.4±0.9 | 71.7±0.9 | 68.8±1.2 | 69.0±1.3 |
| **CD4^+^ T cells**  **(CD3^+^CD4^+^)** |  |  | 58.4±0.6 | 56.6±0.6* | 58.2±0.6 | 57.5±0.6 |
|  | CD38^-^HLA-DR^-^ |  | 35.5±0.7 | 36.9±0.8 | 35.2±0.8 | 36.5±0.8 |
|  | CD38^-^HLA-DR^+^ |  | 3.5±0.2 | 3.8±0.2 | 3.3±0.2 | 3.5±0.2 |
|  | CD38^+^HLA-DR^-^ |  | 58.1±0.9 | 56.3±1.0 | 58.7±1.1 | 57.0±1.1 |
|  | CD38^+^HLA-DR^+^ |  | 2.9±0.2 | 3.1±0.2 | 2.8±0.2 | 3.0±0.2 |
|  | Naïve %  (CD197^+^CD45RA^+^) |  | 53.8±0.9 | 52.6±0.9 | 53.0±1.1 | 52.2±1.1 |
|  |  | CD279^+^ mfi | 102.1±6.5 | 93.7±10.0 | 140.2±10.0 | 123.7±10.0 |
|  | Tem %  (CD197^-^CD45RA^-^) |  | 9.9±0.4 | 10.6±0.4 | 10.3±0.6 | 11.3±0.6 |
|  |  | CD279^+^ mfi | 148.2±6.0 | 137.6±5.9 | 201.0±10.1 | 178.7±10.1 |
|  | Tcm%  (CD197^+^CD45RA^-^) |  | 35.8±0.8 | 35.8±0.8 | 36.4±0.9 | 35.6±0.9 |
|  |  | CD279^+^ mfi | 108.3±5.9 | 92.0±5.9* | 158.5±10.8 | 130.7±10.7^Ψ^ |
|  | Temra %  (CD197^-^CD45RA^+^) |  | 0.40±0.09 | 0.28±0.08 | 0.32±0.07 | 0.25±0.07 |
|  |  | CD279 mfi | 134.3±8.9 | 138.9±8.8 | 186.9±10.5 | 167.3±10.5 |
| **CD8^+^ T cells**  **(CD3^+^CD8^+^)** |  |  | 32.2±0.5 | 32.7±0.5 | 32.5±0.4 | 32.6±0.4 |
|  | CD38^-^HLA-DR^-^ |  | 45.7±1.4 | 46.2±1.4 | 41.5±1.4 | 44.7±1.6 |
|  | CD38^-^HLA-DR^+^ |  | 7.6±0.6 | 7.9±0.6 | 7.1±0.4 | 7.8±0.4 |
|  | CD38^+^HLA-DR^-^ |  | 38.7±1.3 | 35.7±1.3 | 41.5±1.2 | 36.9±1.2** |
|  | CD38^+^HLA-DR^+^ |  | 8.3±0.5 | 10.0±0.6* | 8.9±0.7 | 10.5±0.7^Ψ^ |
|  | Naïve %  (CD197^+^CD45RA^+^) |  | 45.9±1.2 | 41.4±1.2** | 47.4±1.5 | 43.9±1.5^Ψ^ |
|  |  | CD279 mfi | 112.8±7.0 | 114.7±7.0 | 115.6±6.3 | 123.3±6.5 |
|  | Tem %  (CD197^-^CD45RA^-^) |  | 26.2±1.7 | 31.8±1.7* | 27.0±1.6 | 30.0±1.7 |
|  |  | CD279 mfi | 134.2±6.7 | 115.9±6.9* | 196.6±14.2 | 162.4±14.0^Ψ^ |
|  | Tcm%  (CD197^+^CD45RA^-^) |  | 11.3±1.0 | 10.4±1.0 | 12.4±1.2 | 11.0±1.2 |
|  |  | CD279 mfi | 116.4±5.1 | 102.4±5.2* | 160.2±8.4 | 142.3±8.4 |
|  | Temra %  (CD197^-^CD45RA^+^) |  | 15.1±1.1 | 16.0±1.1 | 13.5±0.9 | 14.8±0.9 |
|  |  | CD279 mfi | 158.7±10.7 | 180.8±10.7 | 181.2±12.2 | 204.7±12.3 |
| **T follicular helper (Tfh) CD4^+^** |  |  |  |  |  |  |
|  | Precursor %  (CD45RA^-^CD185^+^CD279^+^CD197^-^) |  | 0.25±0.02 | 0.22±0.02 | 0.89±0.21 | 0.24±0.21* |
|  | Memory %  (CD45RA^-^CD185^+^CD279^+^CD183^-^) |  | 0.44±0.04 | 0.43±0.04 | 0.83±0.12 | 0,58±0.12 |
| **Regulatory T cells**  **(CD3^+^CD4^+^)** |  |  |  |  |  |  |
| **Treg %**  **(CD25^+^CD127^lo/-^)** |  |  | 5.7±0.1 | 5.7±0.1 | 5.3±0.3 | 5.0±0.3 |
|  | Naïve %  (CD45RO^-^) | CD25 mfi | 411.6±5.8 | 409.9±6.0 | 372.3±15.2 | 378.2±15.0 |
|  | Memory  (CD45RO^+^) | CD25 mfi | 585.4±8.6 | 581.4±8.8 | 546.1±24.3 | 558.6±23.9 |
| **T conventional %**  **(CD25^lo/-^CD127^hi^CD194^+^)** |  |  |  |  |  |  |
|  | CD45RO^-^ | CD25 mfi | 14.4±0.8 | 12.2±0.8* | 13.7±0.6 | 14.4±0.7 |
|  | CD45RO^+^ | CD25 mfi | 54.0±2.0 | 52.6±2.1 | 57.4±3.4 | 54.8±3.4 |

Data presented as Least Squares mean ± SEM. ^Ψ^*p*<0.1; **p*<0.05; ***p*<0.01.
