# Supplementary material for: Lactobacillus johnsonii N6.2 Modulates the Host Immune Responses: A Double-Blind, Randomized Trial in Healthy Adults
Source: Front Immunol. 2017 Jun 12;8:655. doi: 10.3389/fimmu.2017.00655 (PMC5466969; doi:10.3389/fimmu.2017.00655)
Supplement: Supplementary file 12 [file Table_6.DOCX]

**Supplementary Table 6.** **Summary of the families that changed in abundance after 8 or 12 weeks of treatment^1^**

|  | **Change in Relative abundance (T8-T0)^1^** | | **Change in Relative abundance (T12-T0)^1^** | | **Change in Relative abundance (T12-T8)^1^** | |
| --- | --- | --- | --- | --- | --- | --- |
| **Family name** | **Placebo** | **Ljo** | **Placebo** | **Ljo** | **Placebo** | **Ljo** |
| ***Lachnospiraceae*** | 0.0030 | 0.0228 | -0.0070 | -0.0009 | -0.0100 | -0.0238 |
| ***Ruminococcaceae*** | -0.0060 | 0.0228* | 0.0051 | 0.0223 | 0.0075 | -0.0005 |
| ***Rikenellaceae*** | 0.0045 | 0.0024 | 0.0010 | 0.0019 | -0.0035 | -0.0005 |
| ***Porphyromonadaceae*** | 0.0065 | -0.0005 | 0.0020 | -0.0023 | -0.0045 | -0.00190 |
| ***Prevotellaceae*** | 0.0205 | -0.0152* | 0.0050 | -0.0071 | -0.0155 | 0.0081 |
| ***unclassified_Mollicutes*** | 0.0010 | 0.0024 |  |  | 0 | -0.0009 |
| ***unclassified__Clostridiales*** | 0.0015 | 0.0007 | 0.0040 | -0.0014 | 0.0005 | -0.0014 |
| ***Streptococcaceae*** | 0.0010 | 0.0014 |  |  | -0.0005 | -0.0019 |
| ***unclassified__Clostridiales2*** | 0.0030 | -0.0014 | 0.0005 | 0.0014 | 0.0001 | 0 |
| ***Enterobacteriaceae*** | 0.0005 | 0.0009 |  |  | -0.0015 | 0.0024 |
| ***Desulfovibrionaceae*** | 0.0001 | 0 |  |  | -0.0030 | 0.0033 |
| ***Halomonadaceae*** | 0.0010 | 0 |  |  | -0.0015 | 0.0005** |
| ***Elusimicrobiaceae*** | 0 | 0.0009 | 0 | 0.0005 | 0 | -0.0005 |
| ***Veillonellaceae*** | -0.0025 | 0.0028 | -0.0010 | 0.0009 | 0.0015 | -0.0019 |
| ***Clostridiaceae*** | -0.0005 | 0.0009 | -0.0020 | 0.0067* | -0.0015 | 0.0057* |
| ***Verrucomicrobiaceae*** | 0.0020 | -0.0014 |  |  | 0.0120 | 0.0147 |
| ***unclassified__Cyanobacteria*** | 0 | 0 | 0 | 0 | 0 | 0 |
| ***Mogibacteriaceae*** | 0 | 0.0005 | 0 | 0 | 0 | -0.0005 |
| ***unclassified__Alphaproteobacteria*** | 0 | 0.0005 | 0 | 0 | 0.0010 | -0.0005 |
| ***Lactobacillaceae*** | -0.0010 | 0.0009 | 0.0005 | 0.0005 | 0.0015 | -0.0005 |
| ***Barnesiellaceae*** | -0.0005 | 0.0005 | -0.0005 | 0.0028 | 0 | 0.0024 |
| ***Erysipelotrichaceae*** | -0.0040 | 0.0033 | 0 | 0 | 0.0005 | -0.0009 |
| ***Christensenellaceae*** | 0 | 0 | -0.002 | 0.0033** | -0.0015 | 0.0033* |
| ***Odoribacteraceae*** | 0.0005 | -0.0014 | 0 | -0.0019 | -0.0005 | -0.0005 |
| ***Paraprevotellaceae*** | 0 | -0.0014 | -0.0005 | 0.0009 | -0.0005 | 0.0024 |
| ***Comamonadaceae*** | -0.0015 | 0 |  |  | 0.0010 | -0.0005 |
| ***Pasteurellaceae*** | -0.0010 | -0.0009 |  |  | 0.0020 | 0.0005 |
| ***unclassified__Streptophyta*** | 0 | -0.0024 |  |  |  |  |
| ***Alcaligenaceae*** | -0.0040 | -0.0024 |  |  | -0.0005 | -0.0029 |
| ***unclassified__Bacteroidales*** | -0.0015 | -0.0057 |  |  |  |  |
| ***Bifidobacteriaceae*** | -0.0075 | -0.0024 | -0.0055 | 0.0033 | 0.0020 | 0.0057 |
| ***Methanobacteriaceae*** | 0.4840 | -0.0033 | -0.0040 | -0.0019 | 0.0035 | 0.0014 |
| ***Bacteroidaceae*** | -0.0045 | -0.0305 | -0.0040 | -0.0450 | 0.0005 | -0.0147 |
| ***Coriobacteriaceae*** |  |  | 0.0010 | 0.0019 | 0.0040 | -0.0009 |
| **Turicibacteraceae** |  |  | 0 | 0 | 0 | 0 |
| ***Peptococcaceae*** |  |  | 0.0005 | 0.0005 | 0.0005 | 0.0005 |
| **Bacteroidales family S24-7** |  |  | 0.0010 | -0.0071 | 0.0025 | -0.0014 |

Data presented as Least Squares mean ± SEM. **p*<0.1; ***p*<0.05; ****p*<0.01.

^1^ Relative change in abundance was calculated as the difference between the mean in abundance at week 8 or 12 and the abundance at week 0.
